# A survey of ontology-enabled processes for dependable robot autonomy

**DOI:** 10.3389/frobt.2024.1377897

**Published:** 2024-07-10

**Authors:** Esther Aguado, Virgilio Gomez, Miguel Hernando, Claudio Rossi, Ricardo Sanz

**Affiliations:** ^1^ Autonomous Systems Laboratory, Universidad Politécnica de Madrid, Madrid, Spain; ^2^ Centre for Automation and Robotics, Universidad Politécnica de Madrid-CSIC, Madrid, Spain

**Keywords:** ontology, robot autonomy, adaptation, robustness, resilience

## Abstract

Autonomous robots are already present in a variety of domains performing complex tasks. Their deployment in open-ended environments offers endless possibilities. However, there are still risks due to unresolved issues in dependability and trust. Knowledge representation and reasoning provide tools for handling explicit information, endowing systems with a deeper understanding of the situations they face. This article explores the use of declarative knowledge for autonomous robots to represent and reason about their environment, their designs, and the complex missions they accomplish. This information can be exploited at runtime by the robots themselves to adapt their structure or re-plan their actions to finish their mission goals, even in the presence of unexpected events. The primary focus of this article is to provide an overview of popular and recent research that uses knowledge-based approaches to increase robot autonomy. Specifically, the ontologies surveyed are related to the selection and arrangement of actions, representing concepts such as autonomy, planning, or behavior. Additionally, they may be related to overcoming contingencies with concepts such as fault or adapt. A systematic exploration is carried out to analyze the use of ontologies in autonomous robots, with the objective of facilitating the development of complex missions. Special attention is dedicated to examining how ontologies are leveraged in real time to ensure the successful completion of missions while aligning with user and owner expectations. The motivation of this analysis is to examine the potential of knowledge-driven approaches as a means to improve flexibility, explainability, and efficacy in autonomous robotic systems.

## 1 Introduction

Autonomous robots have endless possibilities; they are applied in a variety of sectors such as transport, logistics, industry, agriculture, healthcare, education, energy, etc. Robotics has shown enormous potential in diverse tasks and environments. However, there are still open issues that compromise autonomous robot dependability. To support their deployment in real-world scenarios, we need to provide robots with tools to act and react properly in unstructured environments with high uncertainty.

Various strategies support the pursuit of a better grasp of intelligence: neuroscience tries to understand how the brain processes information; mathematics seeks computation and rules to draw valid conclusions using formal logic or handling uncertainty with probability and statistics; control theory and cybernetics aim to ensure that the system reaches desired goals, etc. (Russell and Norvig, 2021). One way that we explore in this survey is the use of symbolic explicit knowledge as a means to enhance system intelligence.

Knowledge representation and reasoning (KR&R) is a sub-area of artificial intelligence that concerns analyzing, designing, and implementing ways of representing information on computers so that computational agents can use this information to derive information implied by it (Shapiro, 2003). Reasoning is the process of extracting new information from the implications of existing knowledge. One of the challenges of getting robots to perform tasks in open environments is that programmers cannot fully predict the state of the world in advance. KR&R provides some background to reason about the runtime situation and act in consequence. In addition to adaptability, these approaches can provide an explanation; knowledge can be queried so humans or other agents can understand why a robot acts in a certain way. Lastly, KR&R provides reusability. The robot needs information about its capabilities and the environment in which it is involved; this information can be shared among different agents, applications, or tasks because knowledge bases can be stored in broadly applicable modular chunks.

To be useable by robots, knowledge bases must be machine-understandable because the robot shall read, reason about, and update its content. In the context of intelligent robots, *ontologies* allow us to define the conceptualizations that the robot requires to support autonomous decision making. These ontologies are written in specific computer languages. In this article, we present a review of the ontologies used by autonomous robots to perform complex missions. In our analysis, we focus on the critical aspects of robot autonomy, that is, how KR&R can contribute to increasing the dependability of these types of systems. We aim to draw a landscape on how ontologies can support decision making to build more dependable robots performing elaborated tasks. The main contributions of this work are:

1. A classification of ontologies based on concepts for autonomous robots,
2. An analysis of existing approaches that use ontologies to facilitate action selection, and
3. A discussion of future research directions to increase dependability in autonomous robots.

This article is structured as follows. Section 2 provides background information on KR&R with a special focus on ontologies for robot autonomy. Section 3 discusses the classification criteria for ontologies that support robust autonomy in robotic applications. In Section 4, we describe the selection process for a systematic review in the field of ontologies for autonomous robots and briefly discuss other surveys in the field. In Section 5, we classify and compare the selected approaches based on their main application domain. Section 6 provides a general discussion and describes future research directions. Lastly, Section 7 presents the article's conclusions.

## 2 Knowledge representation and reasoning for robot autonomy

In this section, we introduce the use of ontologies to increase dependability in autonomous systems and the main languages for knowledge-based agents. Finally, we also discuss the essential and desirable capabilities of fully autonomous robots.

Dependability is the ability to provide the intended services of the system with a certain level of assurance, including factors such as availability, reliability, safety, and security (SEBoK Editorial Board, 2023). As an introductory exploration into this field, Guiochet et al. (2017) provide an analysis of techniques used to increase safety in robots and autonomous systems. Avižienis et al. (2004) offer definitions and conceptualizations of aspects related to dependability and create a taxonomy of dependable computing and its associated faults as a framework.

Dependability ensures consistent performance that goes beyond reliability, as it also considers factors such as fault tolerance, error recovery, and maintaining service levels. Various models of tackling these challenges include fault trees, failure modes and effect analysis (FMEAs), and safety case modes. There are also model-based approaches in which fault analysis can be used in runtime to evaluate goals and develop and execute alternative plans (Abbott, 1990). Similarly, this survey focuses on using explicit models to select the most suitable actions for the situation the robot faces to provide the desired outcomes. This survey concentrates on leveraging ontologies as a means to enhance dependability rather than analyzing dependability aspects concerning robotics.

### 2.1 Languages for ontologies

The most extended approach to robot programming is procedural: The intended robot behavior is explicitly encoded in an imperative language. Knowledge-based agents depart from this, using declarative approaches to abstract the control flow. A knowledge base (KB) stores information that allows the robot to deduce how to operate in the environment. This information can be updated with new facts, and repeated queries are used to deduce new facts relevant to the mission. A successful agent often combines *declarative knowledge* with procedural programming to produce more efficient code (Russell and Norvig, 2021). This declarative knowledge is commonly encoded in logic systems to formally capture conceptualizations.

Prolog (PROgramming in LOGic) is the most widely used declarative programming language. Many knowledge-based systems have been written in this language for legal, medical, financial, and other domains (Russell and Norvig, 2021). Prolog defines a formalism based on decidable fragments of first-order logic (FOL). In addition to its notation, which is somewhat different from standard FOL, the main difference is the closed-world assumption. The closed-world assumption asserts that only the predicates explicitly defined in the KB are true; there is no way to declare that a sentence is false.

Another widely used language for writing ontologies is Ontology Web Language (OWL). OWL is not a programming language like Prolog but rather a family of languages that can be used to represent knowledge. It is designed for applications that need to process information rather than simply presenting information to humans. Therefore, OWL facilitates greater machine interpretability with a panoply of different formats, such as extensible markup language (XML), resource description framework (RDF), and RDF Schema (RDF-S). The language has been specified by the W3C OWL Working Group (2012) and is a cornerstone of the semantic web.

The formal basis of OWL is description logic (DL): a family of languages with a compromise between expressiveness and scalability. DL uses decidable fragments of FOL to reason with more expressiveness. The main difference between Prolog and OWL is that the latter uses the open-world assumption. This means that a statement can be true whether it is known or not; that is, only explicitly false predicates are false, in contrast to the closed-world assumption in which only explicitly true predicates are true.

Regarding accessibility, OWL can be encoded by hand or by editors such as Protégé (Musen, 2015) for a user-friendly environment. OWL can be integrated into robotic software through application program interfaces (APIs) such as Jena Ontology API^1^ or OWLAPI^2^ implemented in Java or OWLREADY (Lamy, 2017) implemented in Python. There are also reasoners such as FaCT++^3^, Pellet^4^>, or HermiT^5^.

Prolog can also be used to reason about knowledge represented by OWL or to directly encode a Prolog ontology. The main Prolog APIs are GNU-PROLOG^6^ and SWI-PROLOG^7^. There is even a package for the robot operating system (ROS) called *rosprolog* ^8^ that interfaces between SWI-Prolog and ROS.

### 2.2 Fundamental and domain ontologies

Ontological systems can be classified according to several criteria. We distinguish three levels of abstraction based on Guarino’s hierarchy (Guarino, 1998): upper level, domain, and application. *Upper-level* or foundational ontologies conceptualize general terms such as object, property, event, state, and relations such as parthood, constitution, participation, etc. *Domain* ontologies provide a formal representation of a specific field that defines contractual agreements on the meaning of terms within a discipline (Hepp et al., 2006); these ontologies specify the highly reusable vocabulary of an area and the concepts, objects, activities, and theories that govern it. *Application* ontologies contain the definitions required to model knowledge for a particular application: information about a robot in a specific environment, describing a particular task. Note that the environment or task knowledge could be a subdomain ontology, depending on the reusability it allows. In fact, the progress from upper-level to application ontologies is a continuous spectrum of concept subclassing, with somewhat arbitrary divisions into levels of abstraction reified as ontologies.

Perhaps the most extended upper-level ontology is the Suggested Upper Merged Ontology (SUMO) (Niles and Pease, 2001; Pease, 2011)^9^. SUMO is the largest open-source ontology that has expressive formal definitions of its concepts. Domain ontologies for medicine, economics, engineering, and many other topics are part of SUMO. This formalism uses the Standard Upper Ontology Knowledge Interchange Format (SUO-KIF), a logical language to express concepts with higher-order logic (a logic with more expressiveness than first-order logic) (Brown et al., 2023).

Another relevant foundational ontology for this research is the Descriptive Ontology for Linguistic and Cognitive Engineering (DOLCE), described as an “ontology of universals” (Gangemi et al., 2002), which means that it has classes but not relations. It aims to capture the ontological categories that underlie natural language and human common sense (Mascardi et al., 2006). The taxonomy of the most basic categories of particulars assumed in DOLCE includes, for example, abstract quality, abstract region, agentive physical object, amount of matter, temporal quality, etc. Although the original version of the few dozen terms in DOLCE was defined in FOL, it has since been implemented in OWL; most extensions of DOLCE are also in OWL.

The DOLCE + DnS Ultralite ontology^10^ (DUL) simplifies some parts of the DOLCE library, such as the names of classes and relations with simpler constructs. The most relevant aspect of DUL is perhaps the design of the ontology architecture based on patterns.

Other important foundational ontologies are the Basic Formal Ontology (BFO) (Arp et al., 2015), the Bunge–Wand–Weber Ontology (BWW) (Bunge, 1977; Wand and Weber, 1993), and the Cyc Ontology (Lenat, 1995). BFO focuses on continuant entities involved in a three-dimensional reality and occurring entities, which also include the time dimension. BWW is an ontology based on Bunge’s philosophical system that is widely used for conceptual modeling (Lukyanenko et al., 2021). Cyc is a long-term project in artificial intelligence that aims to use an ontology to understand how the world works by trying to represent implicit knowledge and perform human-like reasoning.

#### 2.2.1 Robotic domain ontologies

Ontologies have gained popularity in robotics with the growing complexity of actions that systems are expected to perform. A well-defined standard for knowledge representation is recognized as a tool to facilitate human–robot collaboration in challenging tasks (Fiorini et al., 2017). The IEEE Standard Association of Robotics and Automation Society (RAS) created the Ontologies for Robotics and Automation (ORA) working group to address this need.

They first published the Core Ontology for Robotics and Automation (CORA) (Prestes et al., 2013). This standard specifies the most general concepts, relations, and axioms for the robotics and automation domain. CORA is based on SUMO and defines what a robot is and how it relates to other concepts. For this, it defines four main entities: robot part, robot, complex robot, and robotic system. CORA is an upper-level ontology currently extended in the IEEE Standard 1872-2015 (IEEE SA, 2015) with other subontologies, such as CORAX, RPARTS, and POS.

CORAX is a subontology created to bridge the gap between SUMO and CORA. It included high-level concepts that the authors claimed to not be explicitly defined in SUMO and particularized in CORA, in particular those associated with design, interaction, and environment. RPARTS provides notions related to specific kinds of robot parts and the roles they can perform, such as grippers, sensors, or actuators. POS presents general concepts associated with spatial knowledge, such as position and orientation, represented as points, regions, and coordinate systems.

However, CORA and its extensions are intended to cover a broad community, so their definitions of ambiguous terms are based solely on necessary conditions and do not specify sufficient conditions (Fiorini et al., 2017). For this reason, concepts in CORA must be specialized according to the needs of specific subdomains or robotics applications.

CORA, like most of the other application ontologies considered here, is defined in a language of very limited expressiveness, mostly expressible in OWL-Lite, and is therefore limited to simple classification queries. Although it is based on upper-level terms from SUMO, it recreated many terms that could have been used directly from SUMO. Moreover, given its choice of representation language, it did not use the first- and higher-order logic formulas from SUMO, limiting its reuse to only the taxonomy.

The IEEE ORA group created the Robot Task Representation subgroup to produce a *middle-level ontology* with a comprehensive decomposition of tasks, from goal to subgoals, that enables humans or robots to accomplish their expected outcomes at a specific instance in time. It includes a definition of tasks and their properties and terms related to the performance capabilities required to perform them. Moreover, it covers a catalog of tasks demanded by the community, especially in industrial processes (Balakirsky et al., 2017).

This working group also created three additional subgroups for more specific domain knowledge: Autonomous Robots Ontology, Industrial Ontology, and Medical Robot Ontology. Only the first of these has been active. The Autonomous Robot subgroup (AUR) extends CORA and its associated ontologies for the domain of autonomous robots, including, but not limited to, aerial, ground, surface, underwater, and space robots.

They developed the IEEE Standard for Autonomous Robotics Ontology (IEEE SA, 2022) with an unambiguous identification of the basic hardware and software components necessary to provide a robot or a group of robots with autonomy. It was conceived to serve different purposes, such as to describe the design patterns of Autonomous Robotics (AuR) systems, to represent AuR system architectures in a unified way, or as a guideline to build autonomous systems consisting of robots operating in various environments.

In addition to the developments of the IEEE ORA working group, there are other relevant domain ontologies, such as OASys and the Socio-physical Model of Activities (SOMA). The Ontology for Autonomous Systems (OASys) (Bermejo-Alonso et al., 2010) captures and exploits concepts to support the description of any autonomous system with an emphasis on the associated engineering processes. It provides two levels of abstraction systems in general and autonomous systems in particular. This ontology connects concepts such as architecture, components, goals, and functions with the engineering processes required to achieve them.

The SOMA for Autonomous Robotic Agents represents the physical and social context of everyday activities to facilitate accomplishing tasks that are trivial for humans (Beßler et al., 2021). It is based on DUL, extending their concept to different event types such as action, process, and state, the objects that participated in the activities, and the execution concept. It is worth mentioning that SOMA was intended to be used in the runtime along with the concept of narratively enabled episodic memories (NEEMs), which are comprehensive logs of raw sensor data, actuator control histories, and perception events, all semantically annotated with information about what the robot is doing and why using the terminology provided by SOMA.

The relationship between upper-level ontologies and domain ontologies is a relationship of progressive domain focalization (Sanz et al., 1999). The frameworks described in the following sections are mostly specializations of these general robotic ontologies and other foundations.

### 2.3 Capabilities for robot autonomy

Etymologically, autonomy means being governed by the laws of oneself rather than by the rules of others (Vernon, 2014). Beer et al. (2014) provide a definition more closely related to robotics: autonomy as the extent to which a robot can *sense* its environment, *plan* based on that environment, and *act* on that environment with the intention of reaching some task-specific *goal* (either given or created by the robot) without external control. A related, systems-oriented perspective pursued in our lab considers autonomy as a relationship between system, task, and context (Sanz et al., 2000).

Rational agents use sense-decide-act loops to select the best possible action. According to Vernon (2014), cognition allows one to increase the repertoire of actions and extend the time horizon of one’s ability to anticipate possible outcomes. He also reviews several cognitive architectures to support artificial cognitive systems and discusses the relationship between cognition and autonomy. For him, cognition includes six attributes: perception, learning, anticipation, action, adaptation, and, of course, autonomy.

Following the link between cognition and autonomy, Langley et al. (2009) also review cognitive architectures and establish the main functional capabilities that autonomous robots must demonstrate. Knowledge is described as an internal property to achieve the following capabilities: (i) recognition and categorization to generate abstractions from perceptions and past actions, (ii) decision making and choice to represent alternatives for selecting the most prosperous action considering the situation, (iii) perception and situation assessment to combine perceptual information from different sources and provide an understanding of the current circumstances, (iv) prediction and monitoring to evaluate the situation and the possible effects of actions, (v) problem solving and planning to specify desired intermediate states and the actions required to reach them, (vi) reasoning and belief maintenance to use and update the KB in dynamic environments, (vii) execution and action to support deliberative and reactive behaviors, (viii) interaction and communication to share knowledge with other agents, and (ix) remembering, reflection, and learning to use meta-reasoning to use past executions as experiences for the future.

Brachman (2002) argues about the promising capabilities of reflective agents. For him, real improvements in computational agents come when systems know what they are doing, that is, when the agent can understand the situation: what it is doing, where, and why. Brachman establishes practically the same foundations as Langley et al. (2009) but explicitly mentions the necessity of coordinated teams and robust software and hardware infrastructure.

In conclusion, most authors recall the importance of the features described above with different levels of granularity. In the next section, we explain and conceptualize those functional capabilities that enable robot operation autonomously. Note that the systems under study use explicit knowledge—ontologies—as the backbone to achieve autonomy.

## 3 Processes for knowledge-enabled autonomous robots

In this section, we introduce the classification on which we will base the review of ontologies for dependable robot autonomy in Section 5. The classification criterion is based on the capabilities introduced in Section 2.3. It establishes the fundamental processes that an autonomous robot should perform.

### 3.1 Perception

A percept is the belief produced as a result of a perceptor sensing the environment in an instant. Perception involves five entities: sensor, perceived quality, perceptive environment, perceptor, and the percept itself.

A *sensor* is a device that detects, measures, or captures a property in the environment. Sensors can measure one particular aspect of the physical world, such as thermometers, or capture complex characteristics, such as segmenting cameras.

A *perceived quality* is a feature that allows the perceptor to recognize some part of the environment—or the robot itself. Note that this quality is mapped into the percept as an instantiation, a belief produced to translate physical information to the system model. Examples of perceived qualities are the temperature, visual images of the environment, and the rotation of a wheel measured by a rotative encoder placed in a robot.

A *perceptive environment* is the part of the environment that the sensor can detect. The perceptive region can be delimited by the sensor’s resolution or to save memory or other resources.

An agent that perceives is a *perceptor*. It constitutes the link between perception and categorization because it takes sensor information and categorizes it. Usually, the perceptor embodies the sensor, as is the case in autonomous robots, but the two could be decoupled if the system processes information from external sensors. Finally, the *percept* is the inner entity—a belief—that results from the perceptual process.

### 3.2 Categorization

Percepts are instantaneous approximate representations of a particular aspect of the physical world. To provide the autonomous robot with an understanding of the situation in which it is deployed, the perceptor must abstract the sensor information and recognize objects, events, and experiences.

Categorization is the process of finding patterns and categories to model the situation in the robot’s knowledge. It can be done at different levels of granularity. Examples of categorizations appear everywhere: at sensor fusion processes from different types of sensors, with their corresponding uncertainty propagation; at the classification of an entity as a mobile obstacle when it is an uncontrolled object approaching the robot; or, in a more abstract level, when a mobile miner robot recognizes the type of mine ore depending on its geo-chemical properties.

In general, this step corresponds to a combination of information about objects, events, action responses, physical properties, etc., to create a picture of what is happening in the environment and in the robot itself. For this purpose, the robot shall incorporate other processes, such as reasoning and prediction.

### 3.3 Decision making

An autonomous robot must direct its actions towards a goal. When an action cannot be performed, the robot shall implement mechanisms to make decisions and select among the most suitable alternatives for the runtime situation.

Note the difference between decision making and planning. Decision making has a shorter time frame because it focuses on the successful completion of the plan. An example of a decision at this level could be to slightly change the trajectory of a robot to avoid an obstacle and then return to the initial path. Planning, on the other hand, is concerned with achieving a goal; it has a longer time horizon to establish the action sequences to complete a mission. For example, the miner robot presented above must examine the mine, detect the mineral vein, and dig in that direction.

Decision making acts upon the different alternatives that the robot can select, for example, in terms of directions and velocity, but also in terms of what component with functional equivalence can substitute for a defective one. The execution of the action—how it is done—changes slightly, but the plan—the action sequence that achieves the goal—remains the same.

### 3.4 Prediction and monitoring

Once the robot has an internal model, it can supervise the situation. This model can be given *a priori* or created before stating the task; it could also be learned. The model reflects the understanding of the robot about its own characteristics, its interactions with the environment, and the relationships between its actions and its outcomes. At runtime, the robot can use the model to predict the effect of an action. It can also anticipate future events based on the way the situation is evolving.

This mechanism also allows the robot to monitor processes and compare the result obtained with the expected response. In the event of inconsistencies, it can inform an external operator or use adaptation techniques to solve possible errors. For example, if a robot is stuck, it may change its motion direction to get out, and if this is not possible, it can alert the user.

The prediction process can also benefit from a learning procedure to improve the models from experience and refine them over time. Furthermore, monitoring can be used during learning, as it detects errors that can help improve the model.

### 3.5 Reasoning

Reasoning is the process of using existing knowledge to draw logical conclusions and extend the scope of that knowledge. It requires solid definitions and relationships between concepts. It uses instances of such concepts to ground them to the robot’s operation and to be used during the mission.

The reasoning process can be used to infer events based on the current percepts, such as the dynamic object approaching the robot, or to infer the best possible action to overcome it based on its background knowledge, such as reducing speed and slightly changing the direction. Lastly, as robots operate in dynamic worlds—specifically, they operate by changing their environment—the knowledge shall evolve over time.

Reasoning includes two processes: (i) infer and maintain beliefs and (ii) discard beliefs that are no longer valid. Logical systems support assertions and retractions for this purpose; however, they must be handled carefully to maintain ontological consistency. Truth maintenance is a critical capability for cognitive agents situated in dynamic environments.

### 3.6 Planning

Planning is the process of finding a sequence of actions to achieve a goal. To reach a solution, the problem must be structured and well defined, especially in terms of the starting state, which the robot shall transform into a desired goal state. The system also needs to know the constraints to execute an action and its expected outcome, that is, preconditions and postconditions. These conditions are also used to establish the order between actions and the effect that they may have on subsequent actions.

The required information is usually stored in three types of models: environment, robot, and goal models. Most authors only mention the environmental model, which includes the most relevant information about the robot world and its actions, tasks, and goals; however, we prefer to isolate the three models to make explicit the importance of proprioceptive information and performance indicators for a more dependable autonomous robot.

Plans can completely guide the behavior of the robot or suggest a succession of abstract actions that can be expanded in different ways. This can result in branches of possible actions, depending on the result of previous states.

Planning is also closely related to monitoring; the supervision output can conclude the effectiveness of the plan or detect some unreachable planned actions. In this case, the plan may need adjustments, such as changing parameters or replacing some actions. Replanning can use part of the plan or draw a completely new structure depending on the progress and status of the plan and the available components.

Lastly, successful plans or sub-plans can be stored for reuse. These stored plans can also benefit from learning, especially with regard to the environmental response to changes and action constraints and outputs.

### 3.7 Execution

A key process in robotic deployments is the execution of actions that interact with the environment. The robot model must represent the motor skills that produce such changes. Execution can be purely deliberative or combined with more reactive approaches; for example, a patrolling robot may reduce its speed or stop to ensure safety when close to a human—reactiveness—but it also needs a defined set of waypoints to fully cover an area—deliberation.

Hence, an autonomous robot must facilitate the integration of both reactive and deliberative actions within a goal-oriented hierarchy. A strictly reactive approach would limit the ability to direct the robot’s actions toward a defined objective. Meanwhile, an exclusively deliberative approach might be excessively computationally intensive and lead to delayed responses to instantaneous changes.

Another aspect of execution is control. Robots use controllers to overcome small deviations from their state. These controllers can operate in open-loop or closed-loop mode. Open-loop controllers apply predetermined actions based on a set of inputs, assuming that the system will respond predictably. Although they lack the ability to correct runtime deviations, they are often simpler and faster. Closed-loop controllers provide a more accurate and precise action based on inputs and feedback received. Control grounds the decision-making process by specifying the final target value for the robot effectors. It constitutes the final phase of action execution.

### 3.8 Communication and coordination

In many applications, robots operate with other agents—humans or robots of a different nature. Communication is a key feature to organize actions and coordinate them towards a shared goal. Moreover, in knowledge-based systems, communication provides an effective way to obtain knowledge from other agents’ perspectives.

Shared information provides a means to validate perceived elements, fuse them with other sources, and provide access to unperceivable regions of the world. However, this requires a way to exchange information between agents in a neutral, shared conceptualization that is understandable and useable for both.

Once communication is established, we shall coordinate the actions of the systems involved. Decision-making and planning processes should take into account the capabilities and availability of agents to direct and sequence their actions toward the most promising solution. For example, in multirobot patrols, agents shall share their pose and planned path to avoid collisions. Another example could be exploring a difficult-to-access mine in which a wheeled robot could be used for most of the inspection activities, and a legged robot could be used to inspect the unreachable areas.

### 3.9 Interaction and design

Interaction and design are often omitted when analyzing autonomous capabilities. Although they are part of the design phase, this engineering knowledge holds considerable influence over the robot’s performance and dependability.

Interaction between agents can be handled through coordination; however, the embodiment of robots can produce interactions between software and/or hardware components. Robots should be aware of the interaction ports and the possible errors that arise from them. This concern is presented by Brachman (2002), as awareness of interaction allows the robot to step back from action execution and understand the sources of failure. This becomes particularly significant during the integration of diverse components and subsystems, where the application of systems engineering techniques proves to be highly beneficial.

Hernández et al. (2018) argue about the need to exploit functional models to make explicit design decisions and alternatives at runtime. These models can provide background knowledge about requirements, constraints, and assumptions under which a design is valid. With this knowledge, we can endow robots with more tools for adaptability, providing the capability to overcome deviations or contingencies that may occur. For example, a manipulator robot with several tools may have one optimal tool for a task, but if this component is damaged, it can use an alternative tool to solve the problem in a less-than-optimal way.

### 3.10 Learning

Most of the processes described above can improve their efficacy through learning: categorization, decision making, prediction and monitoring, planning, execution, coordination, design, etc. Learning can be divided into three steps: remember, reflect, and generalize.

• *Remember* is the ability to store information from previous executions.
• *Reflect* involves analyzing remembered information to detect patterns and establish relationships.
• *Generalize* is the process of abstract conclusions derived from reflection and subsequently extended to use them in future experiences.

The classification of these competencies reveals their incorporation into KBs, identifies potential underrepresented elements, and explores the contributions of knowledge structures to the decision-making process.

## 4 Review process

One of our main objectives in this article is to conduct a systematic analysis of recent and relevant projects that use ontologies for autonomous robots. The methodology followed is inspired by relevant surveys discussed in the next section, such as those by Olivares-Alarcos et al. (2019) and Cornejo-Lupa et al. (2020). To avoid personal bias, the entire article selection process has been cross-analyzed by two people, and the framework analysis has been validated by the five authors.

The first step in our review process was to search for relevant keywords in scientific databases. Specifically, we used the most extended literature browsers, *Scopus* ^11^ and *Web of Science* ^12^. These databases provide a wide range of peer-reviewed literature, including scientific journals, books, and conference proceedings. Scopus includes more than 7,000 publishers, and WOS includes more than 34,000 journals, including important journals in the field, such as those from IEEE, Springer, and ACM. It also has a user-friendly interface to store, analyze, and display articles. Moreover, related articles cited in the analyzed articles are included in the review process to ensure that relevant articles were not missed. The search was done in terms of title, abstract, or keywords containing the terms robot, ontology, plan, behavior, adapt, autonomy, or fault. In practice, we used the following search string. The search can be replicated using the provided query. However, it is important to note that the survey was conducted in 2022, and there may have been developments or new publications since then. Additionally, a list of the analyzed works and intermediate documents is available upon request.

The required terms are “ontology” and “robot,” as they are the foundation of our survey. Using this restriction may seem somewhat limiting because there could be knowledge-based approaches to cognitive robots that are not based on ontologies. However, in this review, we are specifically interested in using explicit ontologies for this purpose, hence the strong requirement regarding “ontology.” We also target at least one keyword related to (i) selection and arrangement of actions—autonomous, planning, behavior—or (ii) overcoming contingencies—fault, adapt. Note that we use asterisks to be flexible with the notation.

This search returned 695 articles after removing duplicates. To mitigate potential biases, we implemented specific inclusion criteria based on publication dates and citation counts. We only include articles widely cited, with 20 or more citations, before 2010; articles between 2010 and 2015 with five or more citations; and articles between 2015 and 2018 with two or more citations. All articles from 2018 onward were included. With these criteria, we reduced the list to 351 articles. The selection process applied these criteria to identify articles that are not only recent but have also demonstrated impact and influence within their respective publication periods.

Then, we analyzed the content of the articles. We included works in which the main point of the article is the ontology. In particular, the work (i) proposes or extends the ontology and (ii) uses the ontology to select, adapt, or plan actions. We eliminated a number of articles on how to use ontologies to encode simulations, on-line generators of ontologies, or ontologies only used for conversation, perception, or collaboration without impact on robot action. The application of these criteria produced 26 relevant articles.

**FIGURE 1:**
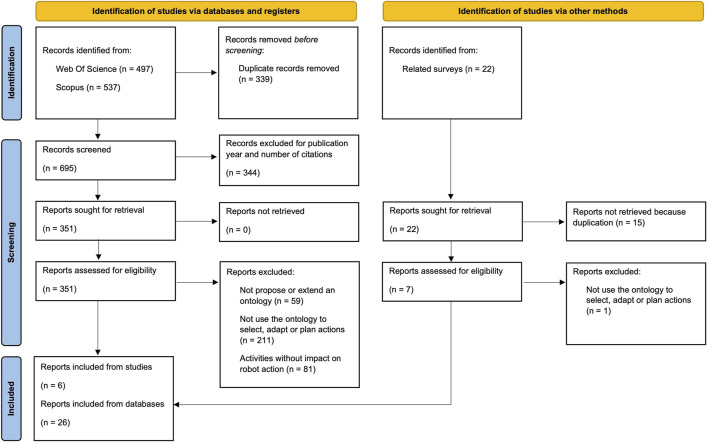
PRISMA flow diagram, adapted from Page et al. (2021).

Finally, we complemented our search with articles from related surveys described in Section 4.1. This ensured that we included all relevant and historical articles in the field with a snowball process. In this step, we obtained 22 more articles; some of them were already included in the previous list, and others did not meet our inclusion criteria. After evaluating them, we included six more articles, resulting in a total of 32 articles in deep review. The entire process is depicted in Figure 1 with a Preferred Reporting Items for Systematic reviews and Meta-Analyses (PRISMA) flow diagram.

### 4.1 Related surveys

This section evaluates a variety of comparative studies and surveys on the application of KR&R to several robotic domains. These works address robot architecture, as well as specific topics such as path planning, task decomposition, and context comprehension. However, our approach transcends these specific domains, aiming to offer a more comprehensive view of the role of ontologies in enhancing robot understanding and autonomy.

Gayathri and Uma (2018) compare languages and planners for robotic path planning from the KR&R perspective. They discuss ontologies for spatial, semantic, and temporal representations and their corresponding reasoning. In Gayathri and Uma (2019), the same authors expand their review by focusing on DL for robot task planning.

Closer to our perspective are articles that compare and analyze different approaches for ontology-based robotic systems, specifically those articles that focus on what to model in an ontology. Cornejo-Lupa et al. (2020) compare and classify ontologies for simultaneous localization and mapping (SLAM) in autonomous robots. The authors compare domain and application ontologies in terms of (i) robot information such as kinematic, sensor, pose, and trajectory information; (ii) environment mapping such as geographical, landmark, and uncertainty information; (iii) time-related information and mobile objects; and (iv) workspace information such as domain and map dimensions. They focus on one important but specific part of robot operation, autonomous navigation, and, in a particular type of robot, mobile robots.

Manzoor et al. (2021) compare projects based on application, ideas, tools, architecture, concepts, and limitations. However, this article does not examine how architecture in the compared frameworks supports autonomy. Their review focuses on objects, environment maps, and task representations from an ontology perspective. It does not compare the different approaches regarding the robot’s self-model. It also does not tackle the mechanisms and consequences of using such knowledge to enhance the robot’s reliability.

Perhaps the most relevant review from our perspective is the one by Olivares-Alarcos et al. (2019). They analyze five of the main projects that use KBs to support robot autonomy. They ground their analysis in (i) ontological terms, (ii) the capabilities that support robot autonomy, and (iii) the application domain. Moreover, they discuss robots and environment modeling. This work is closely related to the development of the recent IEEE Standard 1872.2 (IEEE SA, 2022).

Our approach differentiates itself from previous reviews because we focus on modeling not only robot actions and their environments but also engineering design knowledge. This type of knowledge is not often considered but provides a deeper understanding of the robot’s components and its interaction, design requirements, and possible alternatives to reach a mission. We also base our comparison on explicit knowledge of the mission and how we can ensure that it satisfies the user-expected performance. For this reason, we focus on works that select, adapt, or plan actions. Our search used more flexible inclusion criteria to analyze a variety of articles, even if they address only some of the issues or their ontologies are not publicly available. We have adopted this wide perspective to draw a general picture of different approaches that build the most important capabilities for dependable autonomous robots.

### 4.2 Review scope

Our analysis focuses on exploring the role of ontologies in advancing the autonomy of robotic systems. For this purpose, we delve into research articles that address ontologies created or extended to select, adapt, or plan actions autonomously. We excluded studies that solely encode simulations or utilize ontologies for non-action-related tasks, aiming to concentrate on contributions directly impacting robot action. Furthermore, our scope excludes non-ontology-based approaches, even if they are important for dependability or autonomy, such as all the developments in safe-critical systems.

**FIGURE 2:**
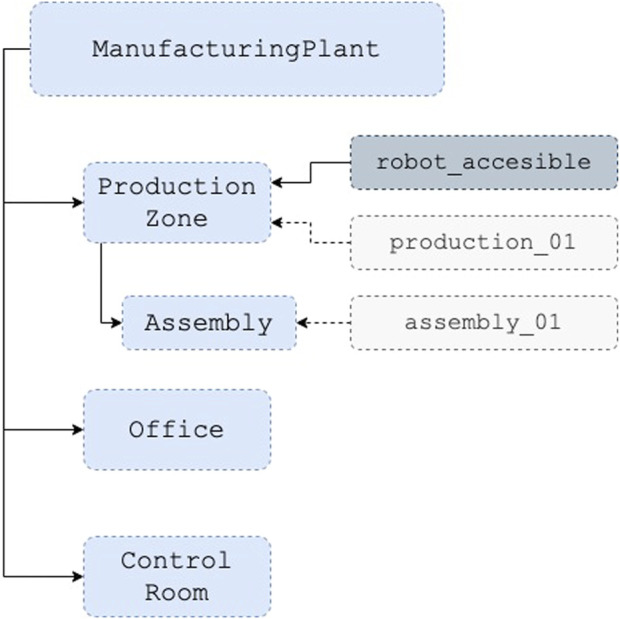
Simple example of manufacturing zones ontology. Classes in light blue, properties in dark blue, and individuals in gray.

An ontology is a shared conceptualization that structures objects into classes, which can be seen as categories within the domain of discourse. While interaction with the world involves specific objects, referred to as individuals or instances, much reasoning occurs at the class level. Classes can possess properties that characterize the collection of objects they represent or are related to other classes. Additionally, they are organized through inheritance, expressed by the subclass relation. Figure 2 represents a simple ontology with partial information about zones in a manufacturing plant.

Consider the assertion of the class ProductionZone with the property robot_accessible. According to this assertion, every instance of ProductionZone, such as the specific area production_01, is considered robot_accessible. In this hierarchy, Assembly is a subclass of ProductionZone. Given this hierarchy, we can deduce that every instance of AssemblyZone inherits the properties of its superclass. Every individual of it, such as assembly_01, inherits the robot_accessible property. Zones without the property, such as Office or Control Room, are considered restricted.

This kind of reasoning based on classification and consistency can be used to derive new assertions about elements in the robot environment and the robot itself. In this example, the robot can use this information to traverse only the accessible areas when selecting a route to traverse the plant. Therefore, the use of knowledge-driven policies facilitates the fulfillment of safety regulations and compliance standards and can enhance human understanding of the decisions the robot is making.

**FIGURE 3:**
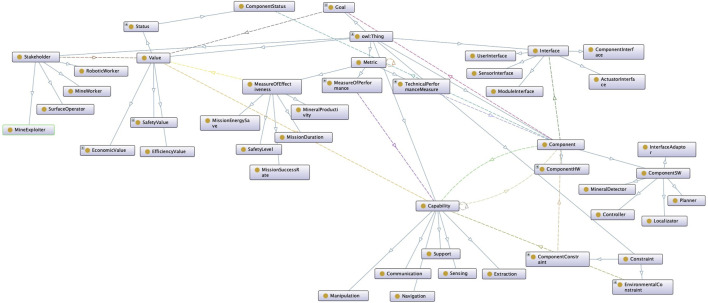
Example of an ontology to formalize robotics system models and their runtime deployment to select the most appropriate reconfiguration strategy in response to unexpected circumstances. This ontology represents the robot components, capabilities, and mission goals. The objective of the ontology is to find adaptation solutions that are not predefined or *ad hoc*; rather, they are computed during operation based on explicit engineering knowledge from the design phase.

If the ontology represented components, capabilities, and goals, these conclusions could be used to adapt the system. Figure 3 represents a robotic ontology based on components, capabilities, goals, and values to determine which design alternatives are available for the robot. Following the approach of TOMASys Hernández et al. (2018), we are working on a system that selects the most suitable reconfiguration action for the robot. For example, if a component fails, the ontology can find another component that provides an equivalent capability so the robot can use it to complete the mission. This KB is also useful for determining which interfaces the system requires in that case, which metrics would be affected, and which stakeholders should be advised of the change. More information on the fundamental aspects of this ontology can be found in Aguado et al. (2024).

The analysis developed here does not address different approaches to solving a specific problem; rather, it focuses on examining, for each framework, the capabilities explicitly represented in ontologies or those that leverage the ontology at runtime to enhance the operation of the robot.

The survey is centered on action-related conceptualizations, with the objective of finding the theoretical foundations and relevant applications of ontological frameworks within the field of robotics. For this reason, and especially given that most of the articles do not provide source files for the ontology, we focus on the concepts, and discussions regarding scalability, computational cost, and efficiency are not within the scope of our study.

### 4.3 Review audience

This survey is directed towards a specific audience that is not seeking introductory knowledge about ontologies or their basic usage. That content can be found in resources such as Staab and Studer (2009). For an introduction from the robotics perspective, refer to Chapter 10 in Russell and Norvig (2021). Our target audience consists of people interested in understanding the practical application of concepts related to robot autonomy during robot task execution. We aim to cater to researchers, practitioners, and academics who already possess a foundational understanding of ontologies and are now seeking to explore how these concepts intersect with and enhance the execution of tasks in robotic systems.

## 5 A survey of applications using ontologies for robot autonomy

The following sections review and compare some projects to find their specific contributions to the field of robotic ontologies. We have organized the analysis of the projects under review based on the application domain—manipulation, navigation, social, and industrial—in which they have been deployed. However, most of the work has a broader perspective and may be applied to other domains. Each analyzed work includes a brief description and a discussion of the above elements. Finally, each domain provides a comparative table with the most relevant aspects of each capability.

### 5.1 Manipulation domain

In the domain of robotics, manipulation refers to the control and coordination of robotic arms, grippers, and other mechanical systems to interact with objects in the physical world. This includes a wide range of applications, from industrial automation to tasks in unstructured environments, such as household chores or healthcare assistance. In this section, most of the work under analysis focuses on domestic applications for manipulation.

#### 5.1.1 KnowRob and KnowRob-based approaches

KnowRob^13^ is a framework that provides a KB and a processing system to perform complex manipulation tasks (Tenorth and Beetz, 2009; Tenorth and Beetz, 2013). KnowRob2^14^ (Beetz et al., 2018) represents the second generation of the framework and serves as a bridge between vague instructions and specific motion commands required for task execution.

KnowRob2’s primary objective is to determine the appropriate action parametrization based on the required motion and identify the physical effects that must be achieved or avoided. For example, if the robot is asked to pick up a cup and pour out its contents, the KB retrieves the necessary action of pouring, which includes a sub-task to grasp the source container. Subsequently, the framework queries for pre-grasp and grasp poses, along with grasp force, to establish the required motion parameters.

KnowRob ontology includes a spectrum of concepts related to robots, including information about their body parts, connections, sensing and action capabilities, tasks, actions, and behavior. Objects are represented with their parts, functionalities, and configuration, while context and environment are also taken into account. Additionally, the ontology incorporates temporal predicates based on event logic and time-dependent relations.

While KnowRob’s initial ontology was based on Cyc (Lenat, 1995), which was designed to understand how the world works by representing implicit knowledge and performing human-like reasoning, Cyc has remained proprietary. OpenCyc, a public initiative related to Cyc, is no longer available. The KnowRob framework later transitioned to DUL, which was chosen for its compatibility with concepts for autonomous robots. KnowRob uses Prolog as a query and assert interface, but all perception, navigation, and manipulation actions are encoded in plans rather than Prolog queries or rules.

One of the main expansions of KnowRob is RoboEarth, a worldwide open-source platform that allows any robot with a network connection to generate, share, and reuse data (Waibel et al., 2011). It uses the principle of linked data connections through web and cloud services to speed up robot learning and adaptation in complex tasks.

RoboEarth uses an ontology to encode concepts and relations in maps and objects and a SLAM map that provides the geometry of the scene and the locations of objects with respect to the robot (Riazuelo et al., 2015). Each robot has a self-model that describes its kinematic structure and a semantic model to provide meaning to robot parts, such as a set of joints that form a gripper. This model also includes actions, their parameters, and software components, such as object recognition systems (Tenorth et al., 2012).

Another example of a KnowRob-based application is Crespo et al. (2018). In this case, the focus is on using semantic concepts to annotate a SLAM map with additional conceptualizations. This application diverges somewhat from KnowRob’s initial emphasis on robot manipulators. The work models the environment utilizing semantic concepts but specifically captures the relationships between rooms, objects, object interactions, and the utility of objects.

The detailed analysis of comparison criteria follows:

• *Perception and categorization:* KnowRob and RoboEarth incorporate inference mechanisms to abstract sensing information, particularly in the context of object recognition (Beetz et al., 2018; Crespo et al., 2018). As highlighted in Tenorth and Beetz (2013), inference processes information perceived from the external environment and abstracts it to the most appropriate level while retaining the connection to the original percepts. These ontologies serve as shared conceptualizations that accommodate various data types and support various forms of reasoning: effectively handling uncertainties arising from sensor noise, limited observability, hallucinated object detection, incomplete knowledge, and unreliable or inaccurate actions (Tenorth and Beetz, 2009).
• *Decision making and planning:* KnowRob places a strong emphasis on determining action parametrizations for successful manipulation, using hybrid reasoning with the goal of *reasoning with eyes and hands* (Beetz et al., 2018).

This approach equips KnowRob with the ability to reason about specific physical effects that can be achieved or avoided through its motion capabilities. Although KnowRob’s KB might exhibit redundancy or inconsistency, the reasoning engine computes multiple hypotheses, subjecting them to plausibility and consistency checks and ultimately selecting the most promising parametrization. The planning component of KnowRob2 is tailored for motion planning and solving inverse kinematics problems. Tasks are dynamically assembled on the basis of the robot’s situation.

• *Prediction and monitoring:* KnowRob uses its ontology to represent the evolution of the state, facilitating the retrieval of semantic information and reasoning (Beetz et al., 2018). Through these heterogeneous processes, the framework can predict the most appropriate parameters for a given situation. However, the monitoring capabilities within this framework are limited to objects in the environment. Unsuccessful experiences are labeled and stored in the robot’s memory, contributing to the selection of action parameters in subsequent scenarios. The framework introduces NEEMs, allowing queries about the robot’s actions, their timing, execution details, success outcomes, the robot’s observations, and beliefs during each action. This knowledge is used primarily during the learning process.
• *Reasoning:* KnowRob2 incorporates a hybrid reasoning kernel comprising four KBs with their corresponding reasoning engines (Beetz et al., 2018):
• Inner World KB: Contains CAD and mesh models of objects positioned with accurate 6D poses, enhanced with a physics simulation.
• Virtual KB: Computed on demand from the data structures of the control system.
• Logic KB: Comprises abstracted symbolic sensor and action data enriched with logical axioms and inference mechanisms. This type of reasoning is the focus of our discussion in this article.
• Episodic Memories KB: Stores experiences of the robotic agent.
• *Execution:* KnowRob execution is driven by competency questions to bridge the gap between undetermined instructions and action. The framework incorporates the Cognitive Robot Abstract Machine (CRAM), where one of its key functionalities is the execution of the plan. The framework provides a plan language to articulate flexible, reliable, and sensor-guided robot behavior. The executor then updates the KB with information about perception and action results, facilitating the inference of new data to make real-time control decisions (Beetz et al., 2010).

RoboEarth and earlier versions of KnowRob rely on action recipes for execution. Before executing an action recipe, the system verifies the availability of the skills necessary for the task and orders each action to satisfy the constraints. In cases where the robot encounters difficulties in executing a recipe, it downloads additional information to enhance its capabilities (Tenorth et al., 2012). Once the plan is established, the system links robot perceptions with the abstract task description given by the action recipe. RoboEarth ensures the execution of reliable actions by actively monitoring the link between robot perceptions and actions (Waibel et al., 2011).

• *Communication and coordination:* RoboEarth (Tenorth et al., 2012) uses a communication module to facilitate the exchange of information with the web. This involves making web requests to upload and download data, allowing the construction and updating of the KB.
• *Learning:* KnowRob includes learning as part of its framework. It focuses on acquiring generalized models that capture how the physical effects of actions vary depending on the motion parametrization (Beetz et al., 2018). The learning process involves abstracting action models from the data, either by identifying a class structure among a set of entities or by grouping the observed manipulation instances according to a specific property (Tenorth and Beetz, 2009). KnowRob2 extends its capabilities with the Open-EASE knowledge service (Beetz et al., 2015), offering a platform to upload, access, and analyze NEEMs of robots involved in manipulation tasks. NEEMs use descriptions of events, objects, time, and low-level control data to establish correlations between various executions, facilitating the learning-from-experience process.

#### 5.1.2 Perception and Manipulation Knowledge

The Perception and Manipulation Knowledge (PMK)^15^ framework is designed for autonomous robots, with a specific focus on complex manipulation tasks that provide semantic information about objects, types, geometrical constraints, and functionalities. In concrete terms, this framework uses knowledge to support task and motion planning (TAMP) capabilities in the manipulation domain (Diab et al., 2019).

PMK ontology is grounded in IEEE Standard 1872.2 (IEEE SA, 2022) for knowledge representation in the robotic domain, extending it to the manipulation domain by incorporating sensor-related knowledge. This extension facilitates the link between low-level perception data and high-level knowledge for comprehensive situation analysis in planning tasks. The ontological structure of PMK comprises a meta-ontology for representing generic information, an ontology schema for domain-specific knowledge, and an ontology instance to store information about objects. These layers are organized into seven classes: *feature*, *workspace*, *object*, *actor*, *sensor*, *context reasoning*, and *actions*. This structure is inspired by OUR-K (Lim et al., 2011), which is further described in Section 5.3).

The detailed analysis of comparison criteria follows:

• *Perception and categorization:* The PMK ontology incorporates RFID sensors and 2D cameras to facilitate object localization, explicitly grouping sensors to define equivalent sensing strategies. This design enables the system to dynamically select the most appropriate sensor based on the current situation. PMK establishes relationships between classes such as *feature*, *sensor*, and *action.* For example, it stores poses, colors, and IDs of objects obtained from images.
• *Decision making and planning:* PMK augments TAMP parameters with data from its KB. The KB contains information on action feasibility considering object features, robot capabilities, and object states. The TAMP module utilizes this information, combining a fast-forward task planner with physics-based motion planning to determine a feasible sequence of actions. This helps determine where the robot should place an object and the associated constraints.
• *Reasoning:* PMK reasoning targets potential manipulation actions employing description logic’s inference for real-time information, such as object positions through spatial reasoning and relationships between entities in different classes. Inference mechanisms assess robot capabilities, action constraints, feasibility, and manipulation behaviors, facilitating the integration of TAMP with the perception module. This process yields information about constraints such as interaction parameters (e.g., friction, slip, maximum force), spatial relationships (e.g., inside, right, left), feasibility of actions (e.g., arm reachability, collisions), and action constraints related to object interactions (e.g., graspable from the handle, pushable from the body).
• *Interaction and design:* PMK represents interaction as manipulation constraints, specifying, for example, which part of an object is interactable, such as a handle. It also considers interaction parameters such as friction coefficient, slip, or maximum force.

#### 5.1.3 Failure Interpretation and Recovery in Planning and Execution

Failure Interpretation and Recovery in Planning and Execution (FailRecOnt)^16^ is an ontology-based framework featuring contingency-based task and motion planning. This innovative system empowers robots to handle uncertainty, recover from failures, and engage in effective human–robot interactions. Grounded in the DUL ontology, it addresses failures and recovery strategies, but it also takes some concepts from CORA and SUMO for robotics and ontological foundations, respectively.

The framework identifies failures through the *non-realized situation* concept and proposes corresponding *recovery strategies* for actions. To improve the understanding of failure, the ontology models terms such as *causal mechanism*, *location*, *time*, and *functional considerations*, which facilitates a reasoning-based repair plan (Diab et al., 2020; Diab et al., 2021).

Failure ontology requires a system knowledge model in terms of *tasks*, *roles*, and *object concepts*. Lastly, FailRecOnt has some similarities with KnowRob; both target manipulation tasks are at least partially based on DUL and share some of the authors. Moreover, they propose using PMK as a model of the system (Diab et al., 2021).

The detailed analysis of comparison criteria follows:

• *Perception and categorization:* Perception in FailRecOnt is limited to action detection to detect abnormal events. For geometric information and environment categorization, the framework leverages PMK to abstract perceptual information related to the environment.
• *Decision making and planning:* FailRecOnt is structured into three layers: planning and execution, knowledge, and an assistant low-level layer. The planning and execution layer provides task planning and a task manager module. The assistant layer manages perception and action execution for the specific robot, determining how to sense an action and checking whether a configuration is collision-free. The framework has been evaluated for a task that involves storing an object in a given tray according to its color; it can handle situations such as facing a closed or flipped box and continuing the plan (Diab et al., 2021).
• *Prediction and monitoring:* Monitoring is a crucial aspect of the FailRecOnt framework. It continuously monitors executed actions and signals a failure to the recovery module if an error occurs. The reasoning component interprets potential failures and, if possible, triggers a recovery action to repair the plan.
• *Reasoning:* FailRecOnt ontology describes how the perception of actions should be formalized. Reasoning selects an appropriate recovery strategy depending on the kind of failure, why it happened, and if other activities are affected, etc.
• *Execution:* FailRecOnt relies on planning for execution. The task planner generates a sequence of symbolic-level actions without geometric considerations. Geometric reasoning comes into play to establish a feasible path. During action execution, the framework monitors each manipulation action for possible failures by sensing. Reasoning is then applied to interpret potential failures, identify causes, and determine recovery strategies.
• *Communication and coordination:* FailRecOnt incorporates the reasoning for communication failure from Diab et al. (2020) to address failures in scenarios where multiple agents exchange information.

#### 5.1.4 Probabilistic Logic Module

Probabilistic Logic Module (PLM) offers a framework that integrates semantic and geometric reasoning for robotic grasping (Antanas et al., 2019). Specific details about the KB, such as the source files, are not publicly available, so the information provided here is derived from articles about the framework.

The primary focus of this work is on an ontology that generalizes similar object parts to semantically reason about the most probable part of an object to grasp, considering object properties and task constraints. This information is used to reduce the search space for possible final gripper poses. This acquired knowledge can also be transferred to objects within the same category.

The object ontology comprises specific objects such as *cup* or *hammer*, along with supercategories based on functionality, such as *kitchen container* or *tool*. The task ontology encodes grasping tasks with objects, such as *pick and place right* or *pour in*. Additionally, a third ontology conceptualizes object-task affordances, considering the manipulation capabilities of a two-finger gripper and the associated probability of success.

In using the ontology, high-level knowledge is combined with low-level learning based on visual shape features to enhance object categorization. Subsequently, high-level knowledge utilizes probabilistic logic to translate low-level visual perception into a more promising grasp planning strategy.

The detailed analysis of comparison criteria follows:

• *Perception and categorization:* This approach is based on visual perception, employing vision to identify the most suitable part of an object for grasping. The framework utilizes a low-level perception module to label visual data with semantic object parts, such as detecting the top, middle, and bottom areas of a cup and its handle. The probabilistic logic module combines this information with the affordances model.
• *Decision making and planning:* PLM uses ontologies to support grasp planning. It integrates ontological knowledge with probabilistic logic to translate low-level visual perception into an effective grasp planning strategy. Once an object is categorized and its affordances are inferred, the task ontology determines the most likely object part to be grasped, thereby reducing the search space for possible final gripper poses. Subsequently, a low-level shape-based planner generates a trajectory for the end effector.
• *Prediction and monitoring:* Although this framework does not specifically predict or monitor robot actions, it uses task prediction to select among alternative tasks based on their probability of success but does not actively monitor them.
• *Reasoning:* PLM uses semantic reasoning to grasp. It selects the best grasping task based on object affordances, addressing the uncertainty of visual perception through a probabilistic approach.
• *Learning:* Learning techniques are used to identify the visual characteristics of the shape, which are then categorized. The robot utilizes the acquired knowledge for grasp planning.

Table 1 provides a concise comparison of the four frameworks analyzed. Although all of them address perception and categorization, decision making and planning, and reasoning, the specific aspects involved depend on the perspective. Only half of the frameworks explicitly utilize their KB for execution, prediction and monitoring, communication and coordination, and learning. In particular, integration and design are addressed exclusively by the PMK framework.

**TABLE 1:** Use of ontologies in the manipulation domain for perception and categorization (P/C), decision making and planning (DM/P), prediction and monitoring (P/M), reasoning (R), execution (E), communication and coordination (C/C), interaction and design (I/D) and learning (L).

	KnowRob (Tenorth and Beetz, 2009; Beetz et al., 2018; Tenorth and Beetz, 2013; Waibel et al., 2011; Tenorth et al., 2012; Crespo et al., 2018; Riazuelo et al., 2015)	PMK (Diab et al., 2019)	FailRecOnt (Diab et al., 2021; Diab et al., 2020)	PLM (Antanas et al., 2019)
P/C	Integrate data types and object recognition	Dynamically select the most appropriate sensor	Action sensing to detect abnormal events	Identify the most suitable part of an object for grasping
DM/P	Action parametrization for successful manipulation	Task and motion planning feasibility	How to sense an action and check if a configuration is collision-free	Translate low-level visual perception into effective grasp planning
P/M	Predict the most appropriate parameters. Monitoring objects in the environment	-	Failure detection and identification of failed recovery attempts	Task prediction to select among alternatives
R	Hybrid, symbolic reasoning for sensor and action data	Integration of task and motion planning with the perception module	How the perception of actions should be formalized	Select the best grasping task based on object affordances
E	Match undetermined instructions and actions	-	Symbolic-level actions without geometric considerations	-
C/C	Web exchange of information	-	Communication failures between multiple agents	-
I/D	-	Information about which part of an object is interactable, constraints	-	-
L	Use previous descriptions of events, objects, time, and low-level control data	-	-	Categorize visual shape features

### 5.2 Navigation domain

The navigation domain describes the challenges and techniques involved in enabling robots to autonomously move around their environment. This involves the processes of guidance, navigation, and control (GNC), incorporating elements such as computer vision and sensor fusion for perception and localization, as well as control systems and artificial intelligence for mapping, path planning, or obstacle avoidance.

#### 5.2.1 Teleological and Ontological Model for Autonomous Systems

Teleological and Ontological Model for Autonomous Systems (TOMASys)^17^ is a metamodel designed to consider the functional knowledge of autonomous systems, incorporating both teleological and ontological dimensions. The teleological aspect includes engineering knowledge, which represents the intentions and purposes of system designers. The ontological dimension categorizes the structure and behavior of the system.

TOMASys serves as a metamodel to ensure robust operation, focusing on mission-level resilience (Hernández et al., 2018). This metamodel relies on a functional ontology derived from the Ontology for Autonomous Systems (OASys) (Bermejo-Alonso et al., 2010), establishing connections between the robot’s architecture and its mission. The core concepts in TOMASys include functions, objectives, components, and configurations. However, it operates as a metamodel and intentionally avoids representing specific features of the operational environment, such as objects, maps, etc.

At the core of the TOMASys framework is the metacontroller. While a conventional controller closes a loop to maintain a system component’s output close to a set point, the metacontroller closes a control loop on top of a system’s functionality. This metacontroller triggers reconfiguration when the system deviates from the functional reference, allowing the robot to adapt and maintain desired behavior in the presence of failures. Explicit knowledge of mission requirements is leveraged for reconfiguration using the system’s functional specifications captured in the ontology.

In practice, TOMASys has been applied to various robots and environments, particularly for navigation tasks. Examples include its application to an underwater mine explorer robot (Aguado et al., 2021) and a mobile robot patrolling a university campus (Bozhinoski et al., 2022).

The detailed analysis of comparison criteria follows:

• *Decision making and planning:* In TOMASys, the metacontrol system manages decision making by adjusting parameters and configurations to address contingencies and mission deviations. It assumes the presence of a nominal controller responsible for standard decisions. In the case of failure detection or unmet mission requirements, the metacontroller selects an appropriate configuration. The planning process is integrated into the metacontrol subsystem, where reconfiguration decisions can impact the overall system plan, potentially altering parameters, components, functionalities, or even relaxing mission objectives to ensure task accomplishment.
• *Prediction and monitoring:* Monitoring is a critical aspect of TOMASys, providing failure models to detect contingencies or faulty components. Reconfiguration is triggered not only in the event of failure but also when mission objectives are not satisfactorily achieved. Observer modules are used to monitor reconfigurable components of the system.
• *Reasoning:* TOMASys uses a DL reasoner for real-time system diagnosis. It propagates component failures to the system level, identifying affected functionalities and available alternatives. This reasoning process helps to select the most promising alternative to fulfill mission objectives.
• *Execution:* The execution in TOMASys follows the monitor-analyze-plan-execute (MAPE-K) loop (IBM Corporation, 2005). It evaluates the mission and system state through monitoring observers, uses ontological reasoners for assessing mission objectives and propagating component failures, decides reconfigurations based on engineering and runtime knowledge, and executes the selected adaptations.
• *Communication and coordination:* TOMASys uses its hierarchical structure to coordinate components working toward a common goal. Components utilize roles that specify parameters for specific functions, and bindings facilitate communication by connecting component roles with function specifications during execution. Bindings are crucial for detecting component failures or errors. In cases where the metacontroller cannot handle errors, a function design log informs the user.
• *Interaction and design:* TOMASys provides a metamodel that leverages engineering models from design time to runtime. This approach aims to bridge the gap between design and operation, relying on functional and component modeling to map mission requirements to the engineering structure. The explicit dependencies between components, roles, and functions, along with specifications of required component types based on functionality, support the system’s adaptability at runtime.

#### 5.2.2 Ontology-based multi-layered robot knowledge framework

The Ontology-based Multi-layered Robot Knowledge Framework (OMRKF) aims to integrate high-level knowledge with perception data to enhance the intelligence of a robot in its environment (Suh et al., 2007). Specific details about the KB, such as the source files, are not publicly available, so the information provided here is derived from articles about the framework.

The framework is organized into knowledge boards, each representing four knowledge classes: perception, activity, model, and context. These classes are divided into three knowledge levels (high, middle, and low). Perception knowledge involves visual concepts, visual features, and numerical descriptions. Similarly, activity knowledge is classified into service, task, and behavior, while model knowledge includes space, objects, and their features. The context class is organized into high-level context, temporal context, and spatial context.

At each knowledge level, OMRKF employs three ontology layers: (a) a meta-ontology for generic knowledge, (b) an ontology schema for domain-specific knowledge, and (c) an ontology instance to ground concepts with application-specific data. The framework uses rules to define relationships between ontology layers, knowledge levels, and knowledge classes.

OMRKF facilitates the execution of sequenced behaviors by allowing the specification of high-level services and guiding the robot in recognizing objects even with incomplete knowledge. This capability enables robust object recognition, successful navigation, and inference of localization-related knowledge. Additionally, the framework provides a querying-asking interface through Prolog, enhancing the robot’s interaction capabilities.

The detailed analysis of comparison criteria follows:

• *Perception and categorization:* OMRKF includes perception as one of its knowledge classes, specifically addressing the numerical descriptor class in the lower-level layer. Examples of these numerical descriptors are generated by robot sensors and image processing algorithms such as Gabor filter or scale-invariant feature transform (SIFT) (Suh et al., 2007).
• *Decision making and planning:* OMRKF uses an event calculus planner to define the sequence to execute a requested service. The framework relies on query-based reasoning to determine how to achieve a goal. In cases of insufficient knowledge, the goal is recursively subdivided into subgoals, breaking down the task into atomic functions such as *go to*, *turn*, and *extract feature.* Once the calculus planner generates an output, the robot follows a sequence to complete a task, such as the steps involved in a delivery mission.
• *Reasoning:* OMRKF employs axioms, such as the inverse relation of *left* and *right* or *on* and *under*, to infer useful facts using the ontology. The framework uses Horn rules to identify concepts and relations, enhancing its reasoning capabilities.

#### 5.2.3 Smart and Networked Underwater Robots in Cooperation Meshes ontology

The Smart and Networked Underwater Robots in Cooperation Meshes (SWARMs) ontology addresses information heterogeneity and facilitates a shared understanding among robots in the context of maritime or underwater missions (Li et al., 2017). Specific details about the KB, such as the source files, are not publicly available, so the information provided here is derived from articles about the ontology.

SWARMs leverages the probabilistic ontology PR-OWL^18^ to annotate the uncertainty of the context based on the multi-entity Bayesian network (MEBN) theory (Laskey, 2008). This allows SWARMs to perform hybrid reasoning on (i) the information exchanged between robots and (ii) environmental uncertainty.

SWARMs establishes a core ontology to interrelate several domain-specific ontologies. The core ontology manages entities, objects, and infrastructures. These ontologies include:

– *Mission and Planning Ontology*: Provides a general representation of the entire mission and the associated planning procedures.
– *Robotic Vehicle Ontology*: Captures information on underwater or surface vehicles and robots.
– *Environment Ontology*: Characterizes the environment through recognition and sensing.
– *Communication and Networking Ontology*: Describes the communication links available in SWARMs to interconnect different agents involved in the mission, enabling communication between the underwater segment and the surface.
– *Application Ontology*: Provides information on scenarios and their requirements. PR-OWL is included in this layer to handle uncertainty.

Li et al. (2017) present an example using SWARMa to monitor chemical pollution based on a probability distribution. SWARMs incorporates this model into the ontology and uses MEBN to deduce the emergency level of the polluted sea region.

The detailed analysis of comparison criteria follows:

• *Perception and categorization:* The SWARMs ontology provides a shared framework to represent the underwater environment. For this reason, it contains classes on sensors and the main concepts of the environment to understand it through its properties, such as water salinity, conductivity, temperature, and currents. Uncertainty reasoning is critical for the categorization of sensor information, especially in harsh maritime and underwater environments.
• *Decision making and planning:* SWARMs uses two levels of abstraction. High-level planning allows the user to describe different tasks related to operations without specifying the exact actions that each robotic vehicle must perform. Low-level planning is performed on each robot to generate waypoints, actions, and other similar low-level tasks.
• *Reasoning:* SWARMs uses a hybrid context reasoner that combines ontological rule-based reasoning with MEBN for probabilistic annotations.
• *Communication and coordination:* A main concern in SWARMs is cooperation; robots share tasks, operations, and actions. The ontology provides transparent information sharing to support the heterogeneity of the data. It also provides an abstraction for communication and networking, describing the communication links available from command control stations to vehicles and from vehicles to command control stations.

#### 5.2.4 Robot Task Planning Ontology

The Robot Task Planning Ontology (RTPO) Sun et al. (2019b) is an effective knowledge representation framework for robot task planning. Specific details about the KB, such as the source files, are not publicly available, so the information provided here is derived from articles about the ontology.

Designed to accommodate temporal, spatial, continuous, and discrete information, RTPO prioritizes scalability and responsiveness to ensure practicality in task planning. The ontology comprises three main components: robot, environment, and task.

– *Robot Ontology*: Comprises hardware and software details, location information, dynamic data, and more. Sensors are explicitly modeled as a subclass of hardware, specifying the measurable aspects of the environment. In an experimental context, the robot’s perception is limited to obstacles.
– *Environment Ontology*: Focuses on location and recognition of humans and objects, the environment map, and information collected from other robots.
– *Task Ontology*: Aims to understand how to decompose high-level tasks into atomic actions and adapt plans when the environment changes.

This multi-ontology approach allows RTPO to capture the details of robot task planning by representing both the robot’s internal state and its interactions with the environment.

The detailed analysis of comparison criteria follows:

• *Perception and categorization:* The robot ontology in RTPO considers sensors as a subclass of hardware, specifying measurable aspects of the environment. In the experiment discussed, the robot’s perception is limited to detecting obstacles.
• *Decision making and planning:* Task planning is performed using a domain and a problem file, as in the Planning Domain Definition Language (PDDL). The algorithm described by Sun et al. (2019b) generates and uses these files.

The task planning process is realized by matching the execution preconditions of atomic actions and their effects on the environment from the initial state to the goal state. With this approach, the task planning module can also be executed by changing the input tasks while considering the present action resources. The plans generated by the task planning algorithm are added to the ontology to improve the efficiency of task planning if the same task needs to be planned again.

• *Reasoning:* The reasoning process in RTPO involves updating and storing the knowledge within the ontology. The scalability of robot knowledge aims to enhance the efficiency of reasoning. Experimental scenarios involving the addition of elements to the indoor environment and corresponding KB instances demonstrate changes in consumed time, particularly affecting knowledge query speed. The authors show real-time performance in an application that involves 52,000 individuals, although the impact on the planning process is not explicitly detailed.
• *Communication and coordination:* This process is not explicitly explained by (Sun et al. 2019b). However, a scenario with three robots and two humans is described. Communication among the three robots is highlighted, emphasizing knowledge sharing. The relationships between these entities can be defined by users or developers based on their requirements. In situations with various robots mapping different rooms and using various sensors, the ontology facilitates linking and adding knowledge to constraints to maintain coherence.
• *Learning:* RTPO incorporates learning by updating and adding the plans generated by the task planning algorithm into the ontology. This iterative process aims to improve the efficiency of future task planning, especially when the same task is encountered again.

#### 5.2.5 Guidance, navigation, and control for planetary rovers

Burroughes and Gao (2016) present an architectural solution to address limitations in autonomous software and GNC structures designed for extraterrestrial planetary exploration rovers. Specific details about the KB, such as the source files, are not publicly available, so the information provided here is derived from articles about the framework.

This framework uses an ontology to facilitate the autonomous reconfiguration of mission goals, software architecture, software components, and the control of hardware components during runtime. To manage complexity, the self-reconfiguration ontology is organized into modules. The base ontology serves as an upper ontology, including modules that delineate logic, numeric aspects, temporality, fuzziness, confidences, processes, and block diagrams. Additional modules describe the functions of software, hardware, and the environment.

The primary focus of this framework is reconfiguration. Once the ontology manager detects an undesired state change through the monitoring process, it activates a safety mode. In this safety mode, the system can execute either a reactive plan or create a new plan based on inferences drawn from the new situation.

Following the replanning process, new mission goals are established, allowing the robot to exit safety mode and resume normal operation. The framework specifically aims to minimize odometry errors and ensure safety during travel. To achieve this objective, the connective architecture and processes, such as navigation, localization, control, mapping, as well as sensors and the locomotion system, can be fine-tuned and optimized according to the environment and faulty hardware conditions. For example, certain methods may prioritize tolerance to sensor noise over absolute accuracy in localization.

The detailed analysis of comparison criteria follows:

• *Decision making and planning:* Burroughes and Gao (2016) integrate a reactive approach with a deliberative layer for quick responses. Precalculated responses are prepared for likely changes, but in the absence of options, the rational agent resorts to deliberative techniques. Ontologies and the PDDL are used for knowledge representation and planning, respectively. Actions correspond to specific configurations of services, with plans defined using the initial state of the world, the goal state, the resources of the system, the safety criteria, and the rules. The approach employs a pruning algorithm to reduce possible actions and planning space.
• *Prediction and monitoring:* The focus is on replanning, requiring monitoring to trigger the process. Generic inspectors perform network, resource, and state checks, and specific inspectors manage tasks like checking camera performance, monitoring, and updating the ontology on the current state of the world. The system can decide the depth of monitoring for each subsystem, balancing computational resources and self-protection.
• *Reasoning:* Reasoning is used to configure the software elements of the rover, update the state of the world, and decide whether the system should re-plan to adapt to changes or use pre-established reactive responses. The ontology checks for knowledge incoherence when adding new information.
• *Execution:* The MAPE-K loop is used for self-reconfiguration. When the monitor detects a change, the ontology provides the knowledge to select how the system should evolve. The configuration of the components is established through planning or reactivity. Navigation and operation components are organized into modular services with self-contained functionality to allow reconfiguration.
• *Communication and coordination:* Communication requirements, such as publication rate, conditions, and effects, are used to establish appropriate communication links between modules, treating communication as a reconfigurable service within the framework.
• *Interaction and design:* While not explicitly detailing the design or requirements, the system reasons in terms of service capacities, considering measures such as accuracy or suitability for sensor noise in sensor processing algorithms. It also incorporates safety criteria to select system changes, providing design and engineering knowledge to select alternative designs based on runtime situations.

#### 5.2.6 Collaborative context awareness in search and rescue missions

Chandra and Rocha (2016) present a framework for collaborative context awareness using an ontology that comprises high-level aspects of urban search and rescue (USAR) missions. Specific details about the KB, such as the source files, are not publicly available, so the information provided here is derived from articles about the framework.

The framework serves as an efficient knowledge-sharing platform to represent and correlate various mission concepts, including those related to agents and their capabilities, scenarios, and teams. The ontology facilitates the sharing of homogeneous knowledge among all robots and supports human–robot collaboration by reasoning based on rules provided by humans.

The detailed analysis of comparison criteria follows:

• *Perception and categorization:* The system employs perceived and pre-processed information gathered from its own sensors, as well as from other agents, such as humans and robots, to continually update its KB. Specifically, it focuses on entities within context classes and their relationships, including information about smoke levels, visibility, location, and temperature.
• *Decision making and planning:* The framework offers an efficient knowledge-sharing strategy that improves decision-making processes. For example, it facilitates the management of a global map with shared events and real-time tracking of agent locations.
• *Reasoning:* In this framework, reasoning plays a crucial role in storing and disseminating information between agents. The KB retains essential details such as location, temperature, visibility, battery status, tasks, and detection of victims or fires. Furthermore, reasoning contributes to resilience against communication failures by storing information for future sharing. This process supports coordination, including the incorporation of new team members or the temporary removal and subsequent reintegration of teammates.
• *Communication and coordination:* Building on a foundation of multirobot cooperation, Chandra and Rocha (2016) structure the framework to represent the team and mission within an ontology. This enables the creation of a global map and the selection of suitable candidates for various tasks. Examples of coordination include telepresence and compensating for a teammate’s immobility. Furthermore, the framework ensures resilience in the face of communication failures. For example, all events detected by a robot are logged in the local KB and shared across the team to synchronize knowledge. In situations of communication loss or isolation from teammates, a list of unattended events is maintained and shared after communication is restored.

#### 5.2.7 Autonomous vehicle situation assessment and decision making

Huang et al. (2019) use ontologies for situation assessment and decision making in the context of autonomous vehicles navigating urban environments. Specific details about the KB, such as the source files, are not publicly available, so the information provided here is derived from articles about the ontologies.

The KB integrates vehicle information, storing details of the permissible *directions* a vehicle can take. Furthermore, the KB includes representations of both static and dynamic obstacles, as well as relevant information on the characteristics of roads, distinguishing between *highways* and *urban roads*. The ontology incorporates specific scenarios that the autonomous vehicle could encounter on the road, such as proximity to an *intersection*, traversing a *bridge*, or executing a *U-turn*. The autonomous driving system leverages this KB to assess the current driving scenario, facilitating well-informed decisions on whether to maintain its current trajectory or initiate a lane change.

The detailed analysis of comparison criteria follows:

• *Decision making and planning:* The decision-making process is tied to situation assessment, employing a methodology that evaluates the safety of the surroundings of the vehicle. This involves characterizing the regions around the car and assigning a binary safety value based on the presence of obstacles.

If a region is considered safe, the vehicle continues in its current lane; otherwise, a lane change is initiated. This decision-making step incorporates statistical indicators that account for velocity. Moreover, the model considers legitimacy by adhering to traffic rules during lane changes and reasonableness by querying the global planning path to identify the next road segment or lane, particularly when approaching intersections. This strategic approach prevents the system from optimizing locally at the expense of compromising the overarching global plan.

• *Reasoning:* The reasoner is used to generate behavioral decisions. It employs rules derived from traffic regulations and driving experiences, taking into account various factors that influence the road, such as speed limits, traffic lights, and the presence of surrounding obstacles.
• *Interaction and design:* Although the framework does not explicitly include concepts as design decisions, it addresses requirements such as legality by incorporating traffic rules and reasonableness. This ensures that the behavior of the lane change aligns with the main goal of global planning, avoiding changes to local optimization that may compromise the broader strategic plan.

#### 5.2.8 Search and rescue scenario

Sun et al. (2019a) introduce an ontology tailored for search and rescue (SAR), enhancing the decision-making capabilities of robots in complex and unpredictable scenarios. Specific details about the KB, such as the source files, are not publicly available, so the information provided here is derived from articles about the ontology. The SAR ontology comprises three interlinked subontologies: an *entity* ontology, an *environment* ontology, and a *task* ontology.

– The *entity* ontology includes various types of robots, including ground, underwater, and air robots. It delineates their constituent parts and specifications in terms of hardware and software aspects.
– The *environment* ontology extends the scope to store most of the elements present in SAR scenarios, including the environment map and objects that shall be recognized. Updated knowledge from the environment can be shared among other robots, although the specific mechanisms are not detailed in the presented use case.
– The *task* ontology encapsulates the task-related knowledge that is necessary for informed decision making. This involves task decomposition and allocation facilitated by a hierarchical structure. The ontology defines four typical tasks: *charge*, *search*, *rescue*, and *recognize.* Additionally, atomic actions are articulated by their effects on the state of the environment. The framework proposes a task planning algorithm that aligns the preconditions of execution with the effects on the environment, utilizing the SAR ontology as a foundational framework.

The detailed analysis of comparison criteria follows:

• *Perception and categorization:* The SAR framework focuses on SLAM and autonomous navigation. The authors use a semantic map, which facilitates the recognition and localization of objects. The acquired information dynamically updates the environment ontology, establishing connections between the SLAM map and the semantic details. Bayesian reasoning enhances the precision of victim positioning, while QR code scanning streamlines the acquisition of semantic information, such as vital signs (e.g., heart rate and blood pressure), in disaster rescue scenarios.
• *Decision making and planning:* decision making in this framework is based on structured queries. It begins by defining tasks and identifying the requisite actions, followed by a comprehensive examination of the action properties, including time constraints, preconditions, and postconditions, to achieve the desired state. The planner then specifies a sequenced set of atomic actions. The program can adopt specific search algorithms for planning or reusing previously established plans.
• *Reasoning:* Before planning, the robot conducts a preliminary assessment to determine the suitability of the task for the current state. If considered appropriate, the planning phase starts, identifying the tasks the robot should execute under the given circumstances.
• *Execution:* The robot systematically executes the sequence of atomic actions. When faced with a previously planned task, the robot can query the task definition, retrieving the corresponding atomic action sequence.

Tables 2 and 3 summarize studies in the navigation domain. All frameworks address decision making, planning, and reasoning, mainly to organize tasks. Most of the works focus on perceiving and categorizing measurable aspects of the environment, along with communication and coordination among different elements and systems. TOMASys, the planetary rover scenario, and the SAR scenario explicitly handle prediction, monitoring, and execution. The use of engineering knowledge—interaction and design—is explicitly addressed in TOMASys, the planetary rover scenario, and the autonomous vehicle scenario. Learning is applied only in RTPO to improve the plan generation process.

**TABLE 2:** Part 1: Use of ontologies in the navigation domain for perception and categorization (P/C), decision making and planning (DM/P), prediction and monitoring (P/M), reasoning (R), execution (E), communication and coordination (C/C), interaction and design (I/D) and learning (L).

	TOMASys (Hernández et al., 2018; Bozhinoski et al., 2022; Aguado et al., 2021)	OMRKF (Suh et al., 2007)	SWARMs (Li et al., 2017)	RTPO (Sun et al., 2019b)
P/C	-	Lower-lever perception features such as numerical descriptors	Represent underwater environment properties and sensors	Specification of measurable aspects of the environment
DM/P	Adjust parameters and configurations to address mission contingencies	Calculus planner to accomplish goals and subgoals	Task-level planning and low-level planning to generate waypoints, actions, etc.	Generate and use domains and problem files to generate plans
P/M	Failure models to detect faulty components and goals not achieved	-	-	-
R	Propagate failures to system level, identify affected functionalities and available alternatives	Geometrical relationships between objects	Hybrid context reasoner: rule-based reasoning and probabilistic annotations	Evaluation of real-time performance in reasoning with 52,000 elements of indoor environments
E	MAPE-K loop to evaluate mission status	-	-	-
C/C	Component coordination	-	Robots share tasks, operations, and actions	Knowledge sharing between three robots and two humans
I/D	Use of metamodels to bridge the gap between design and operation	-	-	-
L	-	-	-	Update and add plans generated by the task planning algorithm back into the ontology

**TABLE 3:** Part 2: Use of ontologies in the navigation domain for perception and categorization (P/C), decision making and planning (DM/P), prediction and monitoring (P/M), reasoning (R), execution (E), communication and coordination (C/C), interaction and design (I/D) and learning (L).

	Planetary rover scenario (Burroughes and Gao, 2016)	USAR scenario (Chandra and Rocha, 2016)	Autonomous vehicle scenario (Huang et al., 2019)	SAR scenario (Sun et al., 2019a)
P/C	-	Pre-processed information from sensors and other intelligent agents	-	Semantic map for recognizing and localizing objects
DM/P	Precalculated solutions for likely changes, combined with deliberative techniques	Management of a global map with shared events and real-time tracking of agent locations	Evaluate the safety of the vehicle’s surroundings with statistical indicators	Definition of tasks and identification of requisite actions and their properties
P/M	Monitor resources and state checks to trigger replanning	-	-	-
R	Configure SW elements, update world state, and decide on replanning	Store and distribute information among agents	Apply rules derived from traffic regulations and driving experiences	Evaluate the suitability of the task for the current state
E	MAPE-K loop for reconfiguration	-	-	Query task definition to retrieve the corresponding atomic action sequence
C/C	Communication requirements between modules	Multirobot cooperation, global map, and selection of suitable candidates for each task	-	-
I/D	Explicit service capacities	-	Addresses requirements such as legality by incorporating traffic rules and reasonableness	-
L	-	-	-	-

### 5.3 Social domain

Social robots usually refer to the interaction between robots and humans in a variety of contexts, such as homes, healthcare, education, entertainment, and public spaces. The goal is to create robots that are not only technically capable but also socially aware and able to interact with humans in a manner that is natural, intuitive, and socially acceptable.

#### 5.3.1 Ontology-based unified robot knowledge

The Ontology-based Unified Robot Knowledge (OUR-K) framework for service robots (Lim et al., 2011) consists of five knowledge classes: features, objects, spaces, contexts, and actions. It takes OMRKF, the concept of layer division, from its predecessor. Although OMRKF was originally evaluated in navigation domains, OUR-K extends its application to social robots operating within domestic environments. Specific details about the KB, such as the source files, are not publicly available, so the information provided here is derived from articles about the framework.

A notable feature of OUR-K is its ability to perform tasks even when provided with incomplete information. The framework incorporates a knowledge description of both the robot and its environment, employing algorithms for knowledge association. This involves logic, Bayesian inference, and heuristics; however, logical inference in OUR-K is specifically limited to performing associations between classes and ontological levels, with no indication of alternative inference mechanisms in the literature.

OUR-K includes mechanisms for object recognition, context modeling, task planning, space representation, and navigation. Regarding action representation, its descriptions are simpler and do not contemplate processes as did its predecessor, OMRKF.

Diab et al. (2018) extend OUR-K with a physics-based manipulation ontology to address the challenges that a motion planner might encounter. An actor class is introduced within the knowledge classes to describe the working constraints of the robot, enhancing the planner’s capability to handle interaction dynamics. In addition, the authors propose a prediction mechanism for the entire OUR-K framework. Instead of relying on inferences, a semantic map is generated for categorizing and assigning manipulation constraints, using reasoning based on logical axioms. This approach is evaluated in the context of a specific manipulation task, where a robot serves a liquid drink contained in a can.

The detailed analysis of comparison criteria follows:

• *Perception and categorization:* OUR-K follows the same approach as OMRKF. Perception is abstracted and stored in the feature, space, and object classes. The feature class defines the same knowledge level as the OMRKF. The space class defines a topological map in the middle level and a semantic map in the higher one. The object class middle level includes the object name and function, whereas the top layer defines generic information and relationships among objects; for example, a cup is a type of container.
• *Decision making and planning:* The OMRKF successor, OUR-K (Lim et al., 2011), uses the same structure based on the abductive event calculus planner to reach a hierarchical abstraction of space elements and behaviors. High-level tasks, such as *delivery*, are decomposed into mid-level sub-tasks, such as *go to goal space*, *find object*, or *generate context.* These sub-tasks are further planned as sequences of primitive behaviors, such as *go to* or *recognize object.* The approach used in Diab et al. (2018) based on OUR-K also integrates the three levels of the action class for planning. The planner consults the topological map to determine which object (or robot) occupies which space, and then the semantic map is used to extract object and robot constraints concerning the action. This results in a sequence of actions that may consider contextual information, particularly at the temporal level.
• *Prediction and monitoring:* OUR-K includes rules for navigation monitoring and missing object recognition tasks. However, monitoring is not treated as a distinct process in this framework; instead, it is represented as rules that use several knowledge classes.
• *Reasoning:* Like OMRKF, OUR-K relies on logical inference to associate classes and ontological levels. The bidirectional links between low-level data and high-level knowledge enable both frameworks to fill in missing information, contributing to mission accomplishment.
• *Execution:* OUR-K execution is plan based, where the robot follows the plan and executes a sequence of actions. However, action knowledge is coupled with all other concepts to represent world environments using features such as sensory-motor coordination, object action complex, and affordances. This approach allows robots to perform actions without explicit planning, potentially enabling reactive behavior when necessary (Lim et al., 2011).
• *Interaction and design:* The OUR-K extension presented by Diab et al. (2018) introduces dynamic interaction, focusing on how robotic controllers should adapt to runtime situations. This extension provides information on how a motion planner should handle dynamic forces when manipulating objects, enhancing the framework’s capability to deal with real-time interactions.

#### 5.3.2 OpenRobots Common Sense Ontology

The OpenRobots Common Sense Ontology (ORO) (Lemaignan et al., 2010) establishes a knowledge processing framework and a common sense ontology tailored for facilitating semantic-rich human–robot interaction environments^19^. This approach focuses on conceptualization but offers flexibility for implementing other cognitive functions simultaneously, such as object recognition, task planning, or reasoning. Cooperation is a crucial aspect in ORO, as it targets human–robot interactions.

ORO ontology is based on OpenCyc. The authors specify in the project Wiki^20^ that it shares most of its concepts with the first version of the KnowRob ontology. It includes categories, such as *spatial thing* or *action,* and more concrete concepts, such as *event* or *book*. The authors evaluated their approach by showing robot objects and asking humans about their properties.

The detailed analysis of comparison criteria follows:

• *Perception and categorization:* The authors use several algorithms, such as common ancestors or calculating the best discriminant to categorize perceptions. Discrimination is an important element in human–robot collaboration. For example, if a user asks the robot to bring the bottle and two bottles are available, it can use its ability to discriminate to select the best option.
• *Reasoning:* ORO provides ontological reasoning, as well as external modules that trigger when an event occurs. External modules are used to provide reactive responses. For example, when a human asks the robot to bring an object, the robot creates an instance of this desire. Statements are stored in different memory profiles, such as long-term and short-term memory. Each profile is characterized by a lifetime that is assigned to the stored facts; when a lifetime ends, the ontology removes the fact.
• *Execution:* The framework integrates CRAM (Beetz et al., 2010) to automatically update the ORO server when an object enters or exits the field of view. Execution is query-based, involving combinations of patterns like *is the bottle on the table?* or filters such as *weight < 150.*
• *Communication and coordination:* ORO is designed as an intelligent blackboard that allows various modules to push or pull knowledge to and from a central repository. This facilitates knowledge sharing among agents. Examples of heterogeneity with shared information include event registration, categorization capabilities, and the existence of different memory profiles.

Each agent has an alternative cognitive model for the other agents with whom it has interacted. When ORO identifies a new agent, it automatically creates a new separate model that can be shared with others. This feature enables the storage and reasoning of different and potentially globally inconsistent models of the world.

#### 5.3.3 Ontology for Collaborative Robotics and Adaptation

Ontology for Collaborative Robotics and Adaptation (OCRA)^21^, as described by Olivares-Alarcos et al. (2022), is a specialized ontology designed to represent relevant knowledge in collaborative scenarios, facilitating plan adaptation. Based on KnowRob (Tenorth and Beetz, 2009; Beetz et al., 2018) and its upper ontology, DUL, OCRA shares the support of its predecessor for the temporal history of the KB through episodic memories.

This work primarily employs FOL definitions to establish a foundation for reliable, collaborative robots, with the aim of enhancing the interoperability and reusability of terminology within this domain. The ontology serves as a tool for the robot to address competency questions, such as identifying ongoing collaborations, understanding current plans and goals, determining the agents involved, and assessing plans before and after adaptation.

This ontology has been qualitatively validated for human–robot cooperation, sharing the task of filling the compartments of a tray. The main asset of OCRA, from our perspective, is its explicit representation of collaboration requirements, including safety considerations and a measure of risks, with the objective of effective plan adaptation.

The detailed analysis of comparison criteria follows:

• *Decision making and planning:* OCRA uses ontological knowledge for dynamic plan adaptation during runtime. For example, if the robot had a plan to fill a certain compartment and it is not empty, it adapts to fill another empty compartment. The ontology also stores the adaptation triggers.
• *Reasoning:* As stated above, this work focuses on answering competency questions related to agents involved in collaboration, their plans, goals, and the adaptation of plans when required.
• *Communication and coordination:* OCRA focuses on collaboration with humans, providing a mechanism to explain its plan adaptation through the ontology. This collaboration is enabled through coordination in terms of the plan to solve the task. Specifically, the robot changes its plan according to variations in the environment, such as an already-filled compartment.
• *Learning:* While not explicitly implemented in the current work, OCRA mentions future plans to incorporate episodic memories for learning tasks. Examples include modeling the preferences of different users or learning the structure of tasks to generalize to new ones.

#### 5.3.4 Intelligent Service Robot Ontology

Intelligent Service Robot Ontology (ISRO) (Chang et al., 2020) introduces an ontology-based model tailored for human–robot interaction. The practical application of this approach is demonstrated through the implementation of a social robot that functions as a medical receptionist in a hospital setting. Specific details about the KB, such as the source files, are not publicly available, so the information provided here is derived from articles about the ontology.

ISRO serves as an abstract knowledge management system designed to comprehend information about agents, encompassing both users and robots, as well as their environment. The ontology establishes connections between user knowledge and the robot’s actions and behaviors, incorporating considerations for the spatial and temporal environment. This integration is facilitated through the Artificial Robot Brain Intelligence (ARBI) framework, which includes a task planner, a context reasoner, and a knowledge manager.

ISRO is not limited to a specific domain because it provides a high-level scheme for dynamic generation and management of information. The ontology is divided into four sub-models:

– *User* ontology to store profile information.
– *Robot* ontology to define the robot type, components, and capabilities.
– *Perception* and *environment* to define objects and their attributes, maps, places, temporal events, and relations such as before, after, etc.
– *Action* ontology to define the actions required to perform a task and the expected events in such situations.

The detailed analysis of comparison criteria follows:

• *Perception and categorization:* The framework uses sensor information to recognize the user involved, their face, gender, and age, and recognize their state, that is, the user’s expression, in its application. It also senses the robot’s pose to guide the user to the medical department. Additionally, the framework is also prepared to handle information related to spaces and objects that was not defined in the experiment.
• *Decision making and planning:* The ARBI framework uses path planning to move the robot, specifically, to guide patients to the correct medical department. It is based on JAM architecture (Huber, 1999), which defines goals and plans using the belief, desire, and intention (BDI) agent (Chang et al., 2020). The path is produced using semantic spatial knowledge, defining the spaces the robot shall traverse and relationships between spaces such as *connected to.* The semantic path is translated into topological waypoints using knowledge about objects and a map. This path is stored in the KB and shared between robots.
• *Reasoning:* The knowledge manager infers spatial and temporal information, such as the relationships of currently recognized objects and the time intervals between events. It also characterizes users. It uses Prolog to recognize dynamically generated information. However, only information considered important or critical is stored in the KB as static information.

#### 5.3.5 Service robot scenario

Ji et al. (2012) provide a flexible framework for service robots using the automatic planner Stanford Research Institute Problem Solver (STRIPS). Specific details about the KB, such as the source files, are not publicly available, so the information provided here is derived from articles about the framework.

The ontology represents two main types of information, environmental description and primitive robot actions, which handle spatial uncertainties of particular objects and the primitive actions that the robot can perform. Actions include four main attributes to comply with STRIPS: precondition, postcondition, input, and result. The planning process is optimized using a recursive back-trace search method and knowledge information to limit the search space. The framework is applied to the Care-O-Bot agent in a scenario in which it shall get a milk box.

The detailed analysis of comparison criteria follows:

• *Perception and categorization:* Symbol grounding is crucial for Ji et al. (2012), as it bridges the gap between abstract planning and actual robot sensing and actuation. For example, a task of moving a table involves moving the robot near the table. The symbol grounding specifies exactly where *“near the table”* is.
• *Decision making and planning:* This framework uses recursive backtracing search for action planning in dynamic environments. The use of semantic maps can improve the search for an object by limiting the search space using semantic inference.
• *Reasoning:* Ji et al. (2012) use reasoning to retrieve information about spatial objects, such as the location of the table where milk is stored. Specific actions, such as *workspace of*, are defined to support reasoning and enable the retrieval of spatial information about an object. For example, a milk box could provide a result of *above the table* or *in the refrigerator*, as it is a perishable product. This framework also provides a likelihood estimation of possible locations of objects.
• *Execution:* Execution uses a central controller to propagate the action to the task planner, the user interface, and the low-level robot modules. Each action is represented as a state machine that is executed in real time. After the action is finished, it updates the models based on the result and the action postcondition.

Table 4 depicts the main aspects of each capability in the social applications reviewed. All capabilities incorporate reasoning using knowledge from the ontology, with the majority also utilizing it for perception and categorization, decision making and planning, and execution. Communication and coordination are only present in two of the five analyzed works. Prediction and monitoring, interaction and design, and learning are the least addressed capabilities across these applications.

**TABLE 4:** Use of ontologies in the social domain for perception and categorization (P/C), decision making and planning (DM/P), prediction and monitoring (P/M), reasoning (R), execution (E), communication and coordination (C/C), interaction and design (I/D) and learning (L).

	OUR-K (Lim et al., 2011, Diab et al., 2018)	ORO (Lemaignan et al., 2010)	OCRA (Olivares-Alarcos et al., 2022)	ISRO (Chang et al., 2020)	Service robot scenario (Ji et al., 2012)
P/C	Same approach as OMRKF	Use common ancestors or the best discriminant to categorize	-	Sensor information to recognize the user involved	Abstract planning information
DM/P	Abductive event calculus planner for hierarchical abstraction	-	Dynamic plan adaptation during runtime	Path planning, semantic maps	Recursive back-trace searching
P/M	Navigation monitoring and missing object recognition tasks	-	-	-	-
R	Bidirectional links between low and high levels to fill in missing information	Ontological reasoning combined with external modules triggered when an event occurs	Answering competency questions for collaboration, plans, goals, and adaptations	Infer spatial and temporal information and user characterization	Information about spatial objects
E	Action knowledge is coupled with all other concepts of the knowledge of models and features	Query-based integrated CRAM to automatically update the ontology	-	-	Central controller to propagate the action to planner, user interface, and lower levels
C/C	-	Intelligent blackboard for knowledge sharing among agents	Collaboration with humans, explainability	-	-
I/D	Dynamic interaction, runtime controller adaptation	-	-	-	-
L	-	-	Envisioned as future work	-	-

### 5.4 Industrial domain

Robots in industrial domains aim to increase efficiency and precision in a variety of tasks. These are often related to manufacturing, production, and other industrial processes. Ontologies in this domain aim to increase flexibility, reduce maintenance, or enhance control and inspection processes.

#### 5.4.1 Robot control for Skilled Execution of Tasks

Robot control for Skilled Execution of Tasks (ROSETTA) constitutes an ontology for robots performing manufacturing tasks^22^. Its origins can be traced to the European projects SIARAS, RoSta, and ROSETTA (Stenmark and Malec, 2015). The core of the ontology is mostly focused on robot devices and their skills. It relies on a component called the knowledge integration framework (KIF) to provide services for robotic ontologies and data repositories (Stenmark and Malec, 2015). Note that this should not be confused with the knowledge interchange format, which is a syntax for FOL. KIF acts as an interface to users. They can specify the task by partially ordering the subgoals in an assembly tree; the framework then establishes the action planning and its schedule with limited resources. KIF also provides an execution structure that generates state machines from skill descriptions and their constraints.

Hoebert et al. (2021) use ROSETTA ontology to control the assembly process of a variety of electronic devices. This work focuses on a world model that represents robotic devices and the skills of ROSETTA. It also relies on the boundary representation (BREP) ontology (Perzylo et al., 2015) to semantically encode geometric entities. This work uses the ontology to conceptualize objects and their properties in the environment. It defines a product as a hierarchy of sub-assemblies and parts. Requirements are also included to determine the correct automated manufacturing.

Merdan et al. (2019) also employ the ROSETTA ontology to control an industrial assembly process, in particular, a pallet transport system and the control of an industrial robot. In this case, it uses the ROSETTA and BREP ontologies to conceptualize the robotic system (e.g., skills, properties, constraints, etc.), the product model (e.g., parts, geometries, assembly orientation, etc.), and the manufacturing infrastructure (e.g., product, storage, sensors, etc.). Rosetta has been defined in OWL.

The detailed analysis of comparison criteria follows:

*Perception and categorization:* The ROSETTA application by Hoebert et al. (2021) provides an object recognition module that links perception data with geometric features represented in the KB.

*Decision making and planning:* The KIF executor of the ROSETTA ontology serves as the planning mechanism. It transforms an assembly graph into a sequence of operations with preconditions and postconditions, subsequently translated into a task state machine. Both Merdan et al. (2019) and Hoebert et al. (2021) approach decision making as a plan generator, utilizing PDDL. Hoebert et al. (2021) provide a generator that extracts information from the ontology to produce the required PDDL files for planning. Merdan et al. (2019) also translate the semantic model, that is, states and actions, into domain and problem files through templates.

*Reasoning:* ROSETTA reasoning is enabled by the KIF server (Stenmark and Malec, 2015). It allows the user to download and upload libraries with object descriptions, task specifications, and skills. The KIF reasoner assists robot programming by retrieving information about tools, sensors, objects, object properties, etc. Hoebert et al. (2021) take advantage of the ROSETTA ontology to select the individual actions and the equipment required to manufacture a product part.

*Execution:* KIF uses state machines to execute the skills defined in the ROSETTA ontology (Stenmark and Malec, 2015). However, Hoebert et al. (2021) and Merdan et al. (2019) use the execution of atomic actions through PDDL commands. With this approach, as discussed by Hoebert et al. (2021), the missing standardization of the meta-level concepts can cause difficulties when integrating existing ontologies and reduce the scalability of the approach. A comparison of the performance using several PDDL planners is discussed in this article. In terms of scalability, Merdan et al. (2019) use a central database server to support a high-performance integration of multiple homogeneous data sources to integrate the ontology with other knowledge sources.

*Communication and coordination:* The ROSETTA-based framework by Merdan et al. (2019) aims to communicate between robots and entities in the external production environment. Additionally, being a component-based approach, it provides infrastructure for intercomponent communication.

*Interaction and design:* ROSETTA provides an engineering specification of workspace objects, skills, and tasks from libraries (Stenmark and Malec, 2015). It acts as a database for all the information present in the object and in the environment. Hoebert et al. (2021) use these design models to establish and verify the manufacturing process, tailoring the requirements to parameters such as assembly operation type, manufacturing constraints, material used, and piece dimensions.

#### 5.4.2 Industrial robotic scenario

Bernardo et al. (2018) define an ontology-based approach for an industrial robotic application focused on inserting up to 56 small pins (sealants) into a harness box terminal specifically tailored for the automotive industry. Specific details about the KB, such as the source files, are not publicly available, so the information provided here is derived from articles about the approach.

The KB is built upon CORA (Prestes et al., 2013) and provides specifications for the robot, the machine vision system, and the tasks required for seamless operation. Task sequences are used to execute the plan, but the machine vision system also uses them to inspect whether the sealants were correctly inserted into the connecting boxes. If not, the system prioritizes applying the sealant in the faulty position(s) in the connecting box.

The detailed analysis of comparison criteria follows:

• *Perception and categorization:* This approach uses vision to inspect robot operations, specifically to verify the correct insertion of sealants in connecting boxes. Image processing is applied for this inspection, although the information is not categorized in the ontology. The framework also utilizes proximity, position, and fiber optic sensors to facilitate robot operation.
• *Decision making and planning:* The framework incorporates visual inspection to validate the precision of the insertion of sealants into the connecting boxes. Additionally, it features a re-planning process that modifies production orders when an error calculus task detects errors. Ontological knowledge is used to establish a sequence of tasks, and in the event of an error, a priority task is immediately added to determine the position of the sealant that requires attention. Once this high-priority task is completed successfully, the robotic system resumes the original production order. The authors define adding this priority task as a re-plan, but we think it corresponds to a planned repair as it returns to the original plan afterward.
• *Reasoning:* This framework uses DL reasoning to acquire valuable data for production and maintenance at the factory level. This reasoning includes information about sensors and actuators and their purposes in various tasks. The knowledge obtained is then utilized to plan and respond to queries, improving the overall decision-making process.

#### 5.4.3 Adaptive agents for manufacturing domains

Borgo et al. (2019) extend the DOLCE ontology from a broad perspective. They validated their approach using a real pilot plant, a reconfigurable manufacturing system designed for recycling printed circuit boards (PCBs). Specific details about the KB, such as the source files, are not publicly available, so the information provided here is derived from articles about the ontology. The ontological framework aims to store knowledge about fundamental assumptions and identify the current scenario, including the presence and location of objects, executed actions, responsible agents, changes occurring, etc.

The KB created by this approach seeks to integrate various perspectives within the enterprise, covering intelligent agents, engineering activities, and management activities. The framework is structured in a deliberative layer to synthesize the actions needed to achieve a goal, an executive and monitoring layer to verify the actions, and a mechatronic system that determines the capabilities of the agent.

• *Decision making and planning:* The updated KB uses an abstraction of the device to be controlled, the environment parameters, and the execution constraints to generate a timeline-based planning model. This model provides the atomic operations that a transportation module can perform, depending on its components and the available collaborators. The timeline incorporates temporal flexibility, allowing for relaxed start and end times. The resulting plan represents the potential evolution of the relevant feature. The framework also supports replanning in case of plan execution failure, generating a new plan based on the current state of the mechatronic system.
• *Prediction and monitoring:* Execution is monitored by comparing action outcomes with respect to the expected state of the system and the environment. The monitor process receives signals about the (either positive or negative) outcome of the execution, for example, from the transportation module, and checks whether the actual status of the mechatronic system complies with the plan.
• *Reasoning:* Two types of reasoning are used in this approach. Low-level reasoning infers information about the internal and local contexts of transport modules, identifying system components and available collaborating agents. High-level reasoning utilizes low-level inferences to extract knowledge about the transportation machine’s functional capabilities, deducing specific internal and local contexts. This knowledge is then used to generate the plan and its control model.
• *Execution:* The execution is based on a knowledge-based control loop (KBCL) presented in Borgo et al. (2019). This loop facilitates monitoring by dynamically representing the robot’s capabilities, internal status, and environmental situation to infer the available functionalities. A reconfiguration phase is activated in case of failure or when new capabilities are added, updating the KB and initiating a new iteration of the overall loop. The executor receives sensor signals and feedback from the transportation module, issuing action commands based on the plan.
• *Communication and coordination:* The framework includes the exchange of information through ontologies, employing commands for sending and receiving interactions with other entities. The concept of *collaborators* represents relationships between the agent (transport module) and any connected entities, such as other transport modules or machines.
• *Interaction and design:* The ontology also models engineering and management activities. The authors use engineering approaches to identify high-level functions to be executed to reach a given goal, explore the difference between the actual state and the desired state, and isolate the changes to be made. They include information on operand integrity, operand qualities, quality relationships, etc. These concepts make explicit engineering facts that are usually not included in robotic approaches that complement knowledge about robot capacities and contexts.

Table 5 provides a summary of three frameworks applied to industrial scenarios. ROSETTA encompasses all capabilities except prediction, monitoring, and learning. Similarly, the adaptive agent for manufacturing domains includes all capabilities except perception, categorization, and learning. Lastly, the remaining industrial scenario only covers decision making, planning, and reasoning, which were required to be included in the review, and perception and categorization.

**TABLE 5:** Use of ontologies in the industrial domain for perception and categorization (P/C), decision making and planning (DM/P), prediction and monitoring (P/M), reasoning (R), execution (E), communication and coordination (C/C), interaction and design (I/D) and learning (L).

	ROSETTA (Stenmark and Malec, 2015; Hoebert et al., 2021; Merdan et al., 2019)	Industrial robotic scenario (Bernardo et al., 2018)	Adaptive agent for manufacturing domains (Borgo et al., 2019)
P/C	Object recognition linking perception data with geometric features	Vision to inspect robot operations and proximity, position, and fiber optic sensors	-
DM/P	Transform an assembly graph into a sequence of operations with preconditions and postconditions to create a task-state machine	Replanning process that modifies production orders when an error calculus task detects them	Generate a timeline-based planning model with atomic operations
P/M	-	-	Compare action outcomes with respect to the expected status of the system and the environment
R	Retrieve information about tools, sensors, objects, object properties, etc.	Reasoning to acquire valuable data for production and maintenance at the factory level	Low-level reasoning for local context and high-level reasoning for the transportation machine’s functional capabilities
E	State machines to execute skills	-	Reconfiguration phase is activated in case of failure or when new capabilities are added
C/C	Communication between robot and external production environment entities and infrastructure for intercomponent communication	-	Commands for sending and receiving interactions with other entities
I/D	Design models to establish and verify the manufacturing process, tailoring requirements to parameters	-	Model engineering and management activities
L	-	-	-

### 5.5 Discussion

In this section, we summarize the most relevant results on the reviewed projects as a whole, independent of the discipline in which they were evaluated, as most aim to be generally applicable to any domain. We also discuss other relevant information on the work reviewed, such as the use of temporal information, the encoding language, or the main application and domain.

As far as *perception and categorization* are concerned, most of the projects focus on situation assessment, providing information about the environment to handle the situation and make decisions accordingly. This part is directly related to reasoning because frameworks such as KnowRob (Tenorth and Beetz, 2009; Beetz et al., 2018), SWARMs (Li et al., 2017), or PLM (Antanas et al., 2019) use probability reasoning to generate knowledge about the environment and its affordances.

All works except ORO (Lemaignan et al., 2010) and OCRA (Olivares-Alarcos et al., 2022) use explicit knowledge for *decision making and planning*. These two focus on answering competency questions related to actions or planning for actions in future developments. Geometric and grasp planning are widely used along with task planners to establish a sequence of atomic actions. PDDL is the most widely used task planner. Replanning is also important for some of these articles: TOMASys (Hernández et al., 2018), RTPO (Sun et al., 2019b), FailRecOnt (Diab et al., 2021), industrial application (Bernardo et al., 2018), adaptive manufacturing application (Borgo et al., 2019), and the planetary rover case (Burroughes and Gao, 2016). In these projects, the objective is to maintain the operation in the presence of faults. Lastly, some works also use ontologies to plan in combination with reactive behaviors, such as OUR-K (Lim et al., 2011), ORO (Lemaignan et al., 2010), and the planetary rover application (Burroughes and Gao, 2016).

*Prediction and monitoring* are less present in the reviewed articles. This activity requires a fully operational KB used during runtime and a deeper understanding of the situation and the autonomous robot. Most of the works monitor only the environment, such as KnowRob (Tenorth and Beetz, 2009; Beetz et al., 2018) and OUR-K (Lim et al., 2011). Others only assess the robot state, such as FailRecOnt (Diab et al., 2021), the manipulation application (Borgo et al., 2019), and the planetary rover application (Burroughes and Gao, 2016). Lastly, TOMASys (Hernández et al., 2018) assesses the robot's state and mission status.

*Reasoning* is addressed in all the works. All use ontologies to at least answer queries and verify ontological consistency, as this was an inclusion requirement for the review. Ontological information is used at runtime to recover from failure in some frameworks such as FailRecOnt (Diab et al., 2021), TOMASys (Hernández et al., 2018), and the planetary rover application (Burroughes and Gao, 2016). Reasoners can download additional required information, such as RoboEarth (Tenorth et al., 2012), which uses web and cloud services, or ROSETTA (Stenmark and Malec, 2015), which uses the KIF server to download and upload libraries.

SWARMs (Li et al., 2017) uses a hybrid context reasoner, combining ontological rule-based reasoning with MEBN theory (Laskey, 2008) for probabilistic annotations. PLM (Antanas et al., 2019) uses a probabilistic logic module for grasping, which combines semantic reasoning with object affordances. KnowRob2 (Beetz et al., 2018) takes a further step and provides a hybrid reasoning kernel that enables physics-based reasoning, flexible data structure reasoning, and a detailed robotic agent experience. However, this use comes at a higher cost, as its computation may yield inconsistencies or inefficient reasoning. For this reason, RTPO (Sun et al., 2019b) targets knowledge scalability and efficient reasoning by conducting a study on the performance of real-time reasoning with 52,000 individuals. However, the authors do not describe how this approach affects the planning process.

Not all frameworks use explicit knowledge during *execution;* some of them only perform the action sequence defined in the plan. Others drive its execution to answer competency questions, such as OCRA (Olivares-Alarcos et al., 2022) and KnowRob (Tenorth and Beetz, 2009; Beetz et al., 2018). KnowRob also uses the CRAM (Beetz et al., 2010) executor to update the KB with information about perception and action results, inferring new data to make control decisions at runtime. ORO (Lemaignan et al., 2010) also uses CRAM to update the server when new objects are detected.

Other approaches use state machines, such as ROSETTA (Stenmark and Malec, 2015), or the MAPE-K loop (IBM Corporation, 2005), such as TOMASys (Hernández et al., 2018) and the planetary rover application (Burroughes and Gao, 2016). Similarly, the manufacturing application of Borgo et al. (2019) presents a knowledge-based control loop to combine execution with monitoring.

Other important processes for autonomous systems are *communication and coordination*. All ontological systems can be used easily to answer queries from a human operator. However, this is not sufficient for reliable autonomy; the ontology can provide transparent information to complete the knowledge. SWARMs (Li et al., 2017) enables an abstraction for communication, networking, and information sharing of heterogeneous data. For this, ORO (Lemaignan et al., 2010) uses an intelligent blackboard that allows other modules to push or pull knowledge to a central repository. RoboEarth (Riazuelo et al., 2015) and ROSETTA (Stenmark and Malec, 2015) obtain knowledge from other sources. RoboEarth defines a communication module for uploading and downloading information from the web, and ROSETTA uses the KIF server to download and upload libraries.

Coordination between agents and components of the agent is critical for autonomous operation. ROSETTA provides an infrastructure for intercomponent communication. Component coordination is explicitly addressed in TOMASys (Hernández et al., 2018), the planetary rover from Burroughes and Gao (2016), and the adaptive manufacturing from Borgo et al. (2019). The USAR application from Chandra and Rocha (2016) targets multirobot cooperation for both information sharing and task coordination with other agents. OCRA (Olivares-Alarcos et al., 2022) also handles task coordination and plan adaptation but only concerns human–robot collaboration.

*Interaction and design* are often not explicitly handled during robot operation. However, awareness of interaction allows the robot to step back from action execution and understand the sources of failure. TOMASys (Hernández et al., 2018) provides a metamodel that benefits from the engineering models used during design time. ROSETTA also uses the engineering specification of workspace objects, skills, and tasks as part of the KB. The planetary rover (Burroughes and Gao, 2016) does not explicitly include any information on design or requirements; however, it reasons in terms of the capacity of the services onboard, using some sort of operational requirement to select among alternatives. Similarly, the autonomous vehicle framework (Huang et al., 2019) handles requirements such as legality using traffic rules or reasonableness to support decision making. The adaptive manufacturing application from Borgo et al. (2019) takes a further step in modeling engineering and management activities. Lastly, with regard to dynamical interactions, the OUR-K extension from Diab et al. (2018) includes this knowledge to support the adaptation of robotic controllers. PMK (Diab et al., 2019) represents the interaction as manipulation constraints.

Given the availability of general learning methods, all the processes described above can improve their effectiveness through *learning*. However, this process is less present in the review. Most articles use a form of case-based learning when storing previous successful plans or using episodic memories to recall past situations, such as CORA (Olivares-Alarcos et al., 2022). Other frameworks, such as PMK (Diab et al., 2019) and PLM (Antanas et al., 2019), use learning as part of a situational assessment to categorize perceptual information.

KnowRob provides the most complete learning mechanics. It learns class structures of entities and identifies manipulation places; in addition, it generates generalized models of the physical effects of actions. KnowRob2 (Beetz et al., 2018) makes the learned information available through the Open-EASE knowledge service (Beetz et al., 2015). This service enables any user to upload, access, and analyze episodic memories of robots performing manipulation tasks.

#### 5.5.1 Other aspects

Table 6 depicts other aspects of the frameworks studied, such as the languages used, the use of temporal conceptualizations, and the foundational ontologies.

**TABLE 6:** Summary of other aspects of the framework: concrete encoding languages used, incorporation of temporal conceptualizations, and whether the framework is built upon other works or utilizes an upper-level ontology.

Framework	Encoding Lang	Temporal	Upper-level Ont
KnowRob (Tenorth and Beetz, 2009; Beetz et al., 2018; Tenorth and Beetz, 2013; Waibel et al., 2011; Tenorth et al., 2012; Crespo et al., 2018; Riazuelo et al., 2015)	OWL, Prolog	Intervals	DUL
PMK (Diab et al., 2019)	OWL, Prolog	-	IEEE-1872.2 Std
FailRecOnt (Diab et al., 2021; Diab et al., 2020)	OWL	-	DUL
PLM (Antanas et al., 2019)	OWL	-	-
TOMASys (Hernández et al., 2018; Bozhinoski et al., 2022; Aguado et al., 2021)	OWL	-	OASys
OMRKF (Suh et al., 2007)	Prolog	Context	-
SWARMs (Li et al., 2017)	OWL	-	PR-OWL
RTPO (Sun et al., 2019b)	OWL, Prolog	-	-
Planetary Rovers Scenario (Burroughes and Gao, 2016)	PDDL, OWL	Time Concept	-
USAR Scenario (Chandra and Rocha, 2016)	OWL	-	-
Autonomous Vehicles Scenario (Huang et al., 2019)	Prolog	-	-
SAR Scenario (Sun et al., 2019a)	OWL	-	-
OUR-K (Lim et al., 2011; Diab et al., 2018)	OWL	Context	OMRKF
ORO (Lemaignan et al., 2010)	OWL	-	-
OCRA (Olivares-Alarcos et al., 2022)	OWL	Intervals	DUL, KnowRob
ISRO (Chang et al., 2020)	OWL, Prolog	Time Concept	OpenCyc
Service Robot Scenario (Ji et al., 2012)	OWL, STRIPS	-	-
ROSETTA (Stenmark and Malec, 2015; Hoebert et al., 2021; Merdan et al., 2019)	PDDL, OWL	-	-
Industrial Robotic Scenario (Bernardo et al., 2018)	OWL	-	CORA
Adaptive Agents (Borgo et al., 2019)	OWL	Intervals	DOLCE

Almost all works use OWL or a combination of OWL and Prolog as the KB language. For planning, most of them use geometric planners, such as PLM (Antanas et al., 2019), along with task planners. In some works, task planners are custom made, such as the service robot from Ji et al. (2012). However, in general, task planners are based on ontological queries, such as the SAR scenario (Sun et al., 2019a), the industrial application (Bernardo et al., 2018), and the adaptive manufacturing of Borgo et al. (2019). The planetary rover (Burroughes and Gao, 2016) and ROSETTA (Stenmark and Malec, 2015) use PDDL directly, while RTPO (Sun et al., 2019b) and SWARMs (Li et al., 2017) use a custom approach similar to PDDL.

Some studies use temporal conceptualizations to address planning dynamics. Most of them rely on an ordered sequence of actions. However, KnowRob (Tenorth and Beetz, 2009; Beetz et al., 2018), OCRA (Olivares-Alarcos et al., 2022), and the adaptive manufacturing approach use time intervals to recall a time frame in which the action is executed. OMRKF (Suh et al., 2007) and OUR-K (Lim et al., 2011) include time as part of the context ontology, and the planetary rover (Burroughes and Gao, 2016) and ISRO (Chang et al., 2020) conceptualize time in their KBs.

## 6 Research directions and conclusion

Considering the projects surveyed, we believe that ontologies are a valuable asset that supports robot autonomy. The frameworks discussed provide an advance towards mission-level dependability, increasing robot reliability and availability. However, there are both unsolved issues and valuable possibilities for further research in this domain.

An unsolved issue is the limited dissemination and convergence of the different ontological approaches. There is still insufficient reuse of existing ontologies and interchangeability or interoperability of the different realization frameworks. In this direction, KnowRob (Tenorth and Beetz, 2009; Beetz et al., 2018) is the most documented and impactful project, as it proves its influence on other projects such as ORO (Lemaignan et al., 2010), FailRecOnt (Diab et al., 2021), and OCRA (Olivares-Alarcos et al., 2022). Ontology convergence is always a challenge that is exacerbated by the variety of ontologies, both vertical—the levels of abstraction—and horizontal—the domains of application. However, efforts must be made to ensure this harmonization, given the fact that robots are not isolated entities but are always part of systems-of-systems that share specific forms of knowledge (Sanz et al., 2021).

We believe that future engineering-grade, knowledge-driven autonomous robot software platforms should also be capable of providing three characteristics: explainability, reusability, and scalability. In the articles under review:

• *Explainability* is enabled in most cases—at least in a shallow form—as most frameworks include query/answer interfaces to provide information on robot operation. However, this explainability is tightly bound to the capability of the human user, who is often a robot operator, to fully grasp the explanation. More broad-spectrum, human-aligned ontologies are needed to support a wider spectrum of users.
• *Reusability* is intrinsically present, given the explicitness of declarative knowledge and the sharing of common backgrounds, some of which are built on previous works or upper-layer ontologies. Efforts should made to harmonize and integrate conceptualizations to effectively reuse ontologies—especially in heterogeneous systems.
• *Scalability* and information handling are also partially addressed in some of the realizations, such as reasoning in RTPO (Sun et al., 2019b) and KnowRob (Tenorth and Beetz, 2009; Beetz et al., 2018). However, the problems of scalability remain, such as when dealing with complex systems-of-systems, when addressing geographical or temporal extensive missions, or when knowledge-based collaboration is required, as is the case of cognitive multirobot systems or human–robot teams.

Therefore, we propose a shared concern in future works in this field to improve the capabilities of robots and contribute to the community in these three aspects. There are already some initiatives, such as CRAM from KnowRob authors, a software toolbox for the design, implementation, and deployment of manipulation activities (Beetz et al., 2010). This toolbox supports planning, beliefs, and KnowRob reasoners and has been used in other frameworks such as ORO (Lemaignan et al., 2010). The same authors provide the package *rosprolog*, a bidirectional interface between SWI-Prolog and ROS, to make this logic language accessible to the main robotics middleware. However, these tools are tailored for KnowRob-based environments and are sometimes not well documented or maintained.

The conclusions we extract from this survey are that most of the work is focused on categorization, decision making, and planning. However, *monitoring and coordination* are critical processes with respect to robot control, especially for robust and reliable operation. These kinds of processes are difficult to assess using reusable implementations because of the enormous variability between applications, context, and intervening agents. We propose the use of *explicit engineering models* to facilitate these processes. They are already partially included in TOMASys (Hernández et al., 2018), ROSETTA (Stenmark and Malec, 2015), the planetary rover scenario (Burroughes and Gao, 2016), and the adaptive manufacturing application from Borgo et al. (2019). We believe that the combination of runtime information with design knowledge can be used during decision making and to drive adaptation to bridge the differences between the expected results of robotic users and the actual robot performance.

## 7 Conclusion

In this article, we have presented an overview of projects that use ontologies to enable robot autonomy. We have systematically searched for works that propose or extend an ontology and use that knowledge to select, adapt, or plan robot actions. We have compared approaches in terms of how the processes that support autonomous operation use and update the supporting ontology. We also briefly discussed applications, languages, and time representation in those works.

Conceptualization provides robots with a path to understanding, which is a powerful tool for achieving robustness and resiliency. We have found that most frameworks do not include explicit engineering knowledge about the robot system itself. Information about robot components and their interaction, design requirements, and alternatives enables both explainability and self-adaptation. Robots are far from meeting user and owner expectations, especially in terms of dependability, efficiency, and efficacy. We have found that task and goal specifications usually lack explicit knowledge of the user-expected performance. This is necessary information to create robots that a user can trust because trust depends on the reliable provision of some user-expected result. For example, this information could include user phenomenological aspects, required safety levels, or energy thresholds that make a task unprofitable.

In conclusion, we believe that explicit knowledge can support autonomous robot operations in unstructured environments. There are still open issues with respect to reliability, safety, and explainability to meet the expectations of researchers and the industry. However, the steps taken by these projects in a variety of domains to enhance autonomy using ontologies have proven how promising this approach is. A strong effort in convergence and harmonization is needed, but this is maximally difficult because the necessary concepts are situated in the realm of the always elusive cognitive robot mind.

## Data Availability

The raw data supporting the conclusion of this article will be made available by the authors, without undue reservation.

## Footnotes

1
https://jena.apache.org/documentation/ontology

2
https://github.com/owlcs/owlapi/wiki

3
http://owl.cs.manchester.ac.uk/tools/fact

4
https://github.com/stardog-union/pellet

5
http://www.hermit-reasoner.com/

6
http://www.gprolog.org/

7
https://www.swi-prolog.org/

8
https://github.com/KnowRob/rosprolog

9
https://www.ontologyportal.org, https://github.com/ontologyportal

10
http://ontologydesignpatterns.org/wiki/Ontology:DOLCE+DnS_Ultralite

11
https://www.scopus.com

12
https://www.webofscience.com/wos/alldb/basic-search

13
http://KnowRob.org

14
https://github.com/KnowRob/KnowRob

15
https://github.com/MohammedDiab1/PMK

16
https://github.com/ease-crc/failrecont

17
https://github.com/meta-control/mc_mdl_tomasys

18
https://www.pr-owl.org

19
http://kb.openrobots.org/

20
https://www.openrobots.org/wiki/oro-ontology

21
https://github.com/albertoOA/know_cra

22
https://github.com/jacekmalec/Rosetta_ontology
